# Identifying Cytokine Motif‐Containing, Immunomodulatory Bacterial Proteins in Human Gut Microbiome

**DOI:** 10.1002/advs.202520332

**Published:** 2026-03-23

**Authors:** Ziyu Wang, Siqi Guo, Jing Li, Qianqian Huang, Jing Ning, Binbin Xia, Xun Lv, Xiang Liu, Zeyu Gao, Jine Li, Longchao Liu, Moshi Song, Jun Wang

**Affiliations:** ^1^ Laboratory of Pathogen Microbiology and Immunology Institute of Microbiology Chinese Academy of Sciences Beijing China; ^2^ University of Chinese Academy of Sciences Beijing China; ^3^ Beijing Key Laboratory for Rheumatism Mechanism and Immune Diagnosis (BZ0135) Department of Rheumatology and Immunology Peking University People's Hospital Beijing China; ^4^ Beijing Key Laboratory for Helicobacter Pylori Infection and Upper Gastrointestinal Diseases (BZ0371) Department of Gastroenterology Peking University Third Hospital Beijing China; ^5^ Laboratory of Microbial Resources and Biotechnology Institute of Microbiology Chinese Academy of Sciences Beijing China; ^6^ State Key Laboratory of Organ Regeneration and Reconstruction, Institute of Zoology Chinese Academy of Sciences Beijing China; ^7^ Beijing Institute For Stem Cell and Regenerative Medicine Beijing China; ^8^ Beijing Key Laboratory of Antimicrobial‐Resistant Pathogen Microbiology and AI‐Empowered Containment Beijing China

**Keywords:** colorectal cancer, cytokine motif‐containing proteins, engineered bacteria, gut microbiota, metagenome

## Abstract

Accumulating evidence emphasizes the importance of microbiota–immune interactions in health and disease development, and identified bacteria‐derived small‐molecule metabolites as well as macromolecules such as peptides and proteins as promising therapeutic approaches. Here, we identify cytokine motif‐containing, immunomodulatory bacterial proteins (CMCPs) as a special category of bacterial proteins in both bacterial genomes and gut metagenomes using Hidden Markov Models (HMMs). We further find eight colorectal cancer‑associated CMCPs differentially enriched in patients or healthy controls. Engineered *E. coli* Nissle 1917 (EcN) expressing selected CMCPs administered to *Apc^min/+^* mice selectively colonize intestinal tumors, deliver functional CMCPs in situ, and elicit significant antitumor immune responses while reducing tumor burden. In vitro, purified CMCPs modulate mouse splenic T cells, bone marrow‑derived macrophages and dendritic cells. Our findings indicate that bacterially encoded CMCPs can directly modulate tumor immunity and serve as microbiota‑derived proteins as candidate immunomodulators, which can further be applied in microbiome‐mediated immune therapies for CRC.

## Graphical abstract

By building and constructing HMM (Upper left, blue), the authors identify CMCPs in bacteria genomes and CRC related metagenomes and enriched CRC‐related CMCPs (Upper right, blue). They analyze sequence and structural similarity of hits (Lower left, green), test function with engineered EcN delivered to tumors in a mouse tumor model (Lower right, pink) and in vitro with mouse immune cells, observing antitumor immune effects (Lower right, yellow).

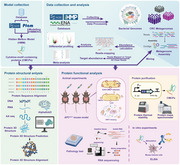

## 1 Introduction

The human microbiome, particularly the gut microbiome, plays an important role in shaping the host immune and metabolic functions, and underlies the occurrence and development of many diseases [1, 2, 3, 4, 5, 6]. The complex gut microbiome produces numerous of metabolites, modulating immune responses and inflammation, including short‐chain fatty acids (SCFAs) that are generally considered anti‐inflammatory, branch‐chained amino acids commonly thought to be pro‐inflammatory, among a growing number of others [7, 8, 9]. On the other hand, macromolecules including DNA, RNA, proteins and polysaccharides from microbes are also highly diverse and have profound impacts on the host immune system, comprising the “pathogen‐associated molecule patterns” (PAMPs) [10]. Within these groups, the large reservoir of bacterial proteins contains proteins that can stimulate inflammatory responses (e.g. superantigens, enterotoxins, proteases etc., from pathogens such as *Staphylococcus aureus* and *Escherichia coli*) [11], those can upregulate oncogenic pathways (e.g., FadA protein from colorectal cancer‐associated *Fusobacterium nucleatum*) [12] and those can induce autoimmune responses by sharing similar sequences to host protein (“molecular mimicry”, as found in rheumatoid arthritis and systemic lupus erythematosus) [13]. Nonetheless, the microbiome also encodes, including but not limited to anti‐inflammatory proteins, antimicrobial peptides (AMPs) against pathogens as well as anticancer peptides (ACPs), which confers protective effects in immune regulation, infection control and cancer contexts [14, 15, 16, 17, 18].

Protein families once believed to be restricted to eukaryotes are increasingly found in bacterial genomes and metagenomes, owing to the expansion of sequencing data and improved analytical approaches. For example, human enzymes participate in the digestion of important peptide hormones, such as dipeptidyl peptidase 4 (DDP4), which cleaves insulin and glucagon‐like peptide‐1, have recently been reported to have homologs in the gut microbiome, with significant contribution to the effects on responses to medication [19]. Components analog to the NOD‐like receptor family pyrin domain containing (NLRP) system that responsible for innate immune responses were also found in bacteria defending against phage infections [20]. Cytokines, another interesting group of eukaryotic immunomodulatory proteins that are considered to be present only in insects and vertebrates, are abundant in mammals [21, 22, 23]. They regulate both innate and adaptive immunity as well as cell growth, differentiation, and death via complex signaling pathways [24, 25, 26, 27]. However, a few cases of cytokine homologs have been reported in viruses, so‐called virokines, including viral interleukin‐10 (vIL‐10) in Epstein‐Barr virus and Cytomegalovirus, which are known to suppress host immune defense and facilitate infections [28, 29]. Although such homologs might derive from horizontal gene transfer, the potential presence of cytokine motif‐containing proteins in the microbiome in general remains an interesting question worth investigating. Here, we define a cytokine motif as a conserved sequence or structural pattern that is characteristic of mammalian cytokines and associated with key functional domains, such as receptor‐binding or immunomodulatory regions.

Identification of cytokine motif‐containing, immunomodulatory bacterial proteins (hereafter referred to as CMCPs) in bacteria would first provide important insights into the roles of gut microbiome‐host cross‐talk in immune modulation and disease development, thereby adding to the current knowledge of microbial molecules affecting host health. More importantly, such CMCPs will greatly expand the repertoire of proteins available for biomedical engineering. For instance, the tumor‐colonizing probiotic *E. coli* Nissle 1917 (EcN) has been engineered to deliver human cytokines as a new approach for tumor immune therapy [30]. Similar bacterial‐based delivery systems, when combined with different cytokines or Immune Checkpoint Inhibitor (ICI) therapies, have potential applications across a broader spectrum of conditions, including autoimmune diseases, metabolic diseases, and cancers. There are, however, obstacles with many human cytokines when expressed in bacteria, including difficulties with low‐expression level, low solubility and lack of post‐translational modification like glycosylation [31, 32, 33, 34]. As a potential solution, CMCPs that retain immunomodulatory activity while being intrinsically compatible with bacterial expression systems, would in theory, mitigate some of those previously mentioned issues and facilitate the development of bacteria‐based immune therapies.

In this study, we constructed Hidden‐Markov models (HMMs) trained on curated alignments of cytokines, enabling the systematic identification of bacterial proteins that share these functionally relevant features. Using these models, we mined bacteria genomes and human gut metagenomes for CMCPs and further identified CMCP groups that were differentially enriched in the metagenome of healthy individuals versus colorectal cancer (CRC) patients. From these candidates, in vivo, the tumor‑colonizing probiotic EcN delivered CMCPs significantly enhanced anti‐tumor immune responses and reduced tumor burden in the *Apc^min/+^* mouse model. While in vitro, a total of four CMCPs were heterologously expressed and demonstrated immunomodulatory effects. These results exhibit a promising applicability of CMCPs for CRC immune therapy.

## 2 Results

### 2.1 Bacterial Genome and Human Microbiome Contain Highly Diverse CMCPs

**FIGURE 1:**
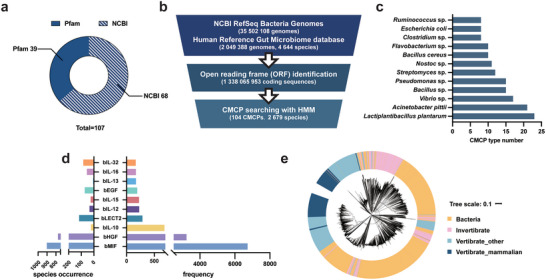
Discovery and distribution of CMCPs in bacterial genomes. (a) Distribution of HMMs collected and constructed in this study. Pfam, dark blue; NCBI, blue with striations. (b) Scheme for mining CMCPs from the bacterial genomes. (c) Species with genomes encoding the largest variety of CMCPs. (d) Occurrence frequency and total numbers of CMCPs in bacterial species. (e) Distribution of bMIF hits across different genome databases. For each bMIF sequence, the tree end points indicate its origin (Bacteria genomes (orange); invertebrate genomes (pink); mammalian vertebrates (dark blue); vertebrate others (light blue)). CMCP, cytokine motif‐containing proteins; EGF, epidermal growth factor; HMM, hidden Malkov model; HGF, hepatocyte growth factor; IL, interleukin; LECT, leukocyte cell‐derived chemotaxin; MIF, macrophage migration inhibitory factor.

To examine the presence of CMCPs in bacterial genomes, we first collected cytokine sequences from NCBI nucleotide sequences (nt) and the Protein Family database (Pfam). Cytokine and chemokine entries were collected from ImmPort Cytokine Registry (totaling 189), in which 39 have established Hidden‐Markov models (HMMs) and have been indexed in the Pfam database (Figure 1a). HMM is a statistical model applied to extract features that are predictive of protein functions based on provided protein sequences. For cytokines entries lacking Pfam models but retained more than 5 sequences in NCBI nt, we built 68 additional HMMs, yielding a total of 107 HMM models for CMCP identification (Figure 1a). Next, we downloaded available bacterial genomes from NCBI (a total of 35 502 108 genomes), and supplemented genomes with a particular focus on the human gut microbiome, those bacterial genomes from Human Reference Gut Microbiome database (HRGM, 204 938 genomes, 4644 species). Open reading frame (ORF) prediction from those genomes identified a total of 1 338 065 953 coding sequences (predicted proteins), which were then screened using our collection of 107 HMMs (Figure 1b).

Our HMM‑based screening resulted in a total of 15 651 predicted proteins with *E‐value* < 0.01, corresponding to a total of 104 human cytokine motifs and hereafter referred to as CMCPs, identified across a total of 2 679 species (mean 5.84 CMCPs per species) (Figure 1b). In terms of types of CMCPs per genome, the largest number was observed in *Lactiplantibacillus plantarum* (23), followed by *Acinetobacter pittii* (21), *Vibrio* sp. (17), *Bacillus* sp. (15), and *Pseudomonas* sp. (15) (Figure 1c). Notably, *Lactiplantibacillus plantarum* can also be found in the human gut microbiome and has been reported as a potentially beneficial therapeutic agent in CRC [35, 36, 37]. Among these CMCPs, those identified to be analogs of macrophage migration inhibitory factor (MIF) (named bMIF to indicate its bacterial origin; similarly for other CMCPs below) have the highest occurrence (6 725, 44.45%) and are detected in 902 species, followed by b‐hepatocyte growth factor (bHGF) (3 232, 21.36%) and found in 778 species. Overall, a total of 17 CMCP families had more than 100 occurrences (Figure 1d).

For those 17 CMCP families, we computed the sequence similarity to their corresponding human cytokine and found that overall, the sequence similarity ranged between 2.63% to 85.29% (mean 19.62%). Additionally, we mined all available genomes of animals from NCBI (6 117 010 genomes, 3 421 926 from vertebrate assemblies, and the rest from invertebrate assemblies). Phylogenetic trees were constructed for those mined cytokines if possible, indicating that the CMCPs overall formed a distinct cluster compared to that of animals, especially to those from vertebrate animals (Figure 1e, Figure S1a–k).

To strengthen the validity of our findings, we searched human proteomics data for the presence of CMCPs. A set of CMCPs was identified, with bMIF and bHGF ranking as the top two most frequently detected CMCPs, similar to our observations in bacterial genomes (Figure S2). This proteomics data confirms that these CMCPs are encoded within microbial genomes and indeed expressed within the human gut microbiome, further validating their potential functional roles in the host‐microbe interaction.

In short, we identified a highly diverse set of CMCPs in bacterial genomes forming a unique collection of sequences, highlighting the large sequence diversity of microbial proteins and a large reservoir of potentially functional microbial genes.

### 2.2 Differential Enrichment of CMCPs in Colorectal Cancer Patients vs. Healthy Controls

**FIGURE 2:**
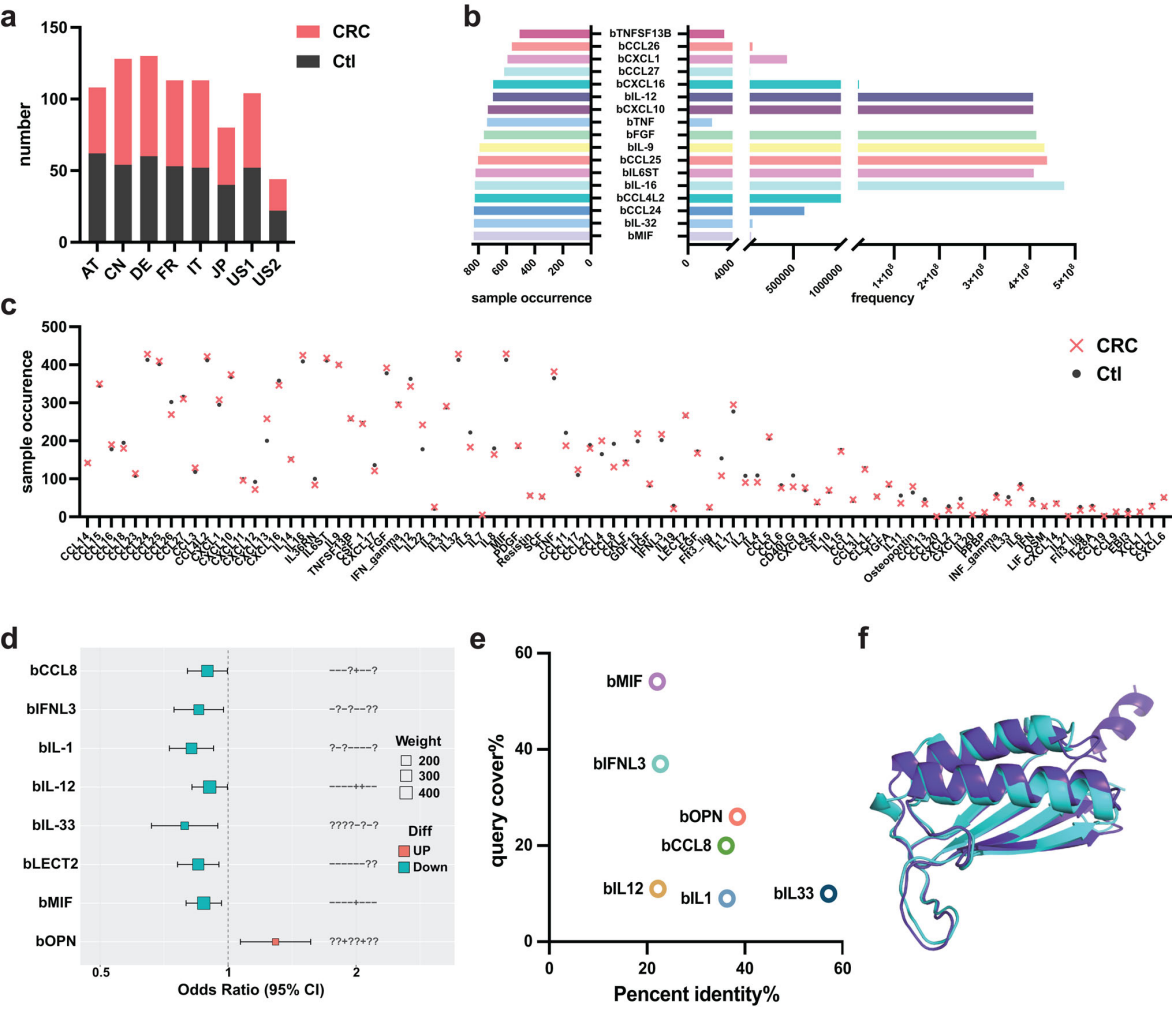
Occurrence, enrichment, and structural comparison of CRC‐related CMCPs. (a) The proportion and sources of the metagenomic data collected from CRC cohorts included in this study. CRC patients, blush pink; Healthy controls, dark grey; AT, Austria; CN, China; FR, France; DE, Germany; IT, Italy; JP, Japan; US, USA. (b) Occurrence frequency and total numbers of CMCPs in CRC‐related metagenomes. (c) CMCP occurrence in all CRC samples. CRC patients, blush pink; Healthy controls, dark grey. (d) Enrichment results for meta‐analysis of CRC‐related metagenome mining of CMCPs (Only significant CMCPs included). X‑axis indicates odds ratio; horizontal error bars represent the 95% confidence intervals. The Y‑axis lists the eight enriched cytokines. Red boxes denote higher abundance of the cytokine homologs in colorectal cancer (CRC) patients, whereas blue boxes denote lower abundance in CRC patients (i.e., relatively enriched in healthy controls). Box area represents the enrichment weight of the homologs. Symbols on the right “+”, “−”, “?” indicate this CMCP is significantly enriched in CRC patients, significantly depleted in CRC patients (enriched in healthy controls), or not significant, respectively, in each cohort. Cohorts are ordered left to right as follows: PRJDB4176, PRJEB10878, PRJEB12449, PRJEB27928, PRJEB6070, PRJEB7774, PRJNA397219, and PRJNA447983. (e) Sequence similarity of enriched CMCP representatives to their corresponding human sequences. (f) Comparison of 3D structures of bMIF with hMIF (Pymol‐align). bMIF, purple; hMIF, cyan. CCL4L2, C‑C motif chemokine ligand 4‑like 2; CLCF1, cardiotrophin‑like cytokine factor 1; CRC, colorectal cancer; Ctl, control; CSF, colony‑stimulating factor; CXCL, C‑X‑C motif chemokine ligand; EBI3, Epstein–Barr virus induced 3; EGF, epidermal growth factor; FGF, fibroblast growth factor; FLT3 ligand, FMS‑like tyrosine kinase 3 ligand; HGF, hepatocyte growth factor; HMM, hidden Markov model; IFN, interferon; IFNL3, interferon lambda 3; IL, interleukin; IL36RN, interleukin 36 receptor antagonist; IL6ST, interleukin‑6 signal transducer; LECT2, leukocyte cell‑derived chemotaxin 2; LIF/OSM, leukemia inhibitory factor/oncostatin M; MIF, macrophage migration inhibitory factor; PDGF, platelet‑derived growth factor; PPBP, pro‑platelet basic protein (CXCL7); SCF, stem cell factor; TGFA, transforming growth factor alpha; TNF, tumor necrosis factor; TNFSF13B, tumor necrosis factor (ligand) superfamily member 13B; XCL, X‑C motif chemokine ligand.

We next examined whether CMCPs present in the human gut microbiome are associated with a representative gastrointestinal disease, colorectal cancer (CRC). To this end, we gathered gut metagenomic data of 306 CRC patients and 316 healthy controls from eight publicly accessible cohorts across seven countries (Austria, AT; China, CN; France, FR; Germany, DE; Italy, IT; Japan, JP; USA, US) (Figure 2a). For each sample we performed metagenomic assembly, predicted open reading frames (ORFs), and screened predicted proteins against the cytokine HMM profiles described above. A total of 92 CMCP families were found in these cohorts. Consistent with the genome‑based survey mentioned above, bMIF is the most abundant CMCP in the metagenome (842 occurrences in all 8 cohorts), followed by bIL‐32 and b‐chemokine (C‐C motif) ligand 24 (bCCL24) (both 841 occurrences in all cohorts) (Figure 2b). Per individual, between 0.37 to 1.53 CMCPs were found present in the gut microbiome, with no statistically significant difference in CMCP occurrences in CRC patients than in healthy individuals (Figure 2c).

Consequently, we performed enrichment analysis across the eight studies. Within each cohort, the relative abundances of CMCPs were first normalized by DESeq2 to eliminate the effects of sequencing depth. Then meta‐analysis of all 89 identified CMCPs was conducted using METAL, which accounts for both the differences between CRC and healthy control as well as cohort size (Figure S3). Using a meta‐*p* value of 0.05 as a cut‐off, we identified a total of seven CMCPs that were significantly enriched in the healthy individuals: b‐C‐C motif chemokine ligand 8 (bCCL8), b‐interferon lambda 3 (bIFNL3), b‐interleukin‐1 (bIL‐1), b‐interleukin‐12 (bIL‐12), b‐interleukin‐33 (bIL‐33), b‐leukocyte cell‐derived chemotaxin 2 (bLECT2), and b‐macrophage migration inhibitory factor (bMIF), while b‐osteopontin (bOPN) was significantly enriched in CRC patients thus depleted in healthy individuals (Figure 2d). To facilitate downstream comparisons, for each differentially enriched CMCP, we selected a representative protein sequence (Table S1) based on maximal length similarity with its corresponding human cytokine counterpart. On average, we found that the eight CMCPs had 33.59% sequence similarity to human analogs, with maximum similarity between bIL‐1 and human IL‐1 (57.14%) and lowest between bMIF and human MIF (22.06%) (Figure 2e). To further assess whether these similarities reflect conservation of intact cytokine domains, we performed HMM‐based domain analysis using curated Pfam models for each corresponding human cytokine (Table S2). The results indicate that only bIFNL3 and bMIF showed near full‐length domain matches, whereas the remaining CMCPs exhibited either partial domain similarity or low‐complexity region overlap. In line with this observation, structures predicted by Alphafold3 revealed that bMIF showed the most similar structure, at a Root Mean Square Deviation (RMSD) of 1.191 Å with its corresponding human analog (Figure 2f). While for other CMCPs, structural prediction resulted in a low predicted Local Distance Difference Test score (pLDDT) (Figure S4) and higher RMSD between 5.23 and 9.91 Å. To conclude, we identified a number of CMCPs that are differentially enriched in healthy individuals or CRC patients. And they share relatively low sequence similarity and moderate to low structural similarity compared to human cytokines.

### 2.3 *E. coli* Nissle 1917‐Delivered CMCPs Modulate Tumor Development in *Apc^**min/+**^* Mice

**FIGURE 3:**
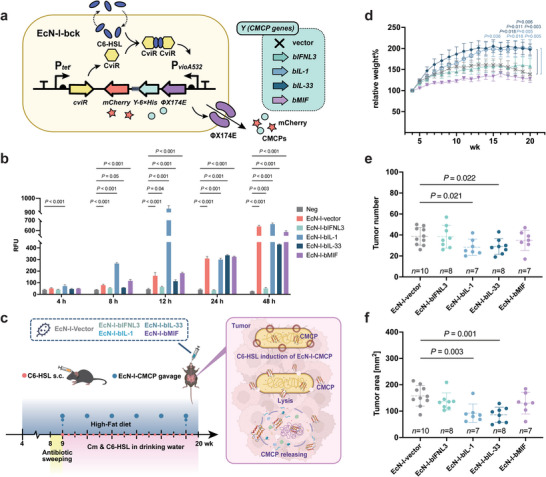
EcN‐delivered CMCPs modulate tumor development in *Apc^min/+^* mice. (a) A schematic diagram of pCRCTS1‐I‐bck plasmid in EcN induced by C6‐HSL. (b) Characterization after induction of EcN‐I‐CMCP. After being induced by C6‐HSL, supernatants were collected, and relative fluorescence units (RFU) levels were measured. Data are presented as mean ± SD. Statistical analysis was performed by two‑way ANOVA (factors: treatment and time) followed by multiple comparisons using Dunnett's test. Significance is indicated directly in the figures with exact *p* values. (c) The timeline and schematic diagram of mice experiment. (d) Percentage changes in body weight of *Apc^min/+^* mice treated with engineered bacteria. Data are presented as mean ± SEM. Statistical analysis was performed by two‑way ANOVA (factors: treatment and time) followed by multiple comparisons using Dunnett's test. Significance is indicated directly in the figures. (e) Tumor number of *Apc^min/+^* mice treated with engineered bacteria. Data are presented as mean ± SD. *n* indicated the number of samples. Statistical analysis was performed by one‑way ANOVA followed by multiple comparisons using Fisher's LSD test. Significance is indicated directly in the figures with exact *p* values. (f) Tumor area of *Apc^min/+^* mice treated with engineered bacteria. Data are presented as mean ± SD. *n* indicated the number of samples. Statistical analysis was performed by one‑way ANOVA followed by multiple comparisons using Dunnett's test. Significance is indicated directly in the figures with exact *p* values.

To elucidate their function, we examined the ability of these CMCPs to affect colorectal cancer development in vivo. We utilized the *E. coli* Nissle 1917 (EcN) system based on its ability to preferentially colonize within tumors. We constructed an inducible lysis system based on quorum‐sensing components, achieving expression and release of CMCPs in EcN cells upon induction by the autoinducer N‑hexanoyl‑L‑homoserine lactone (C6‐HSL) (Figure 3a) [38]. Briefly, this system contains a quorum‐sensing transcription factor CviR, which responds to C6‐HSL, activates the *P_vioA_* promoter and upregulates the downstream transcription of a phage‐derived lysis gene *X17E*. For validation, we engineered a genetic construct for the simultaneous, independent expression of the lysis gene *X17E*, the *CMCP* gene of interest, and the *mCherry* reporter gene under C6‐HSL induction. The detection of fluorescence indicates successful CMCP expression and cell lysis (Figure 3b, Figures S5 and S6). Among the seven CMCPs that were significantly enriched in healthy individuals and depleted in CRC patients, we could successfully express bIFNL3, bIL‐1, bIL‐33, and bMIF, while bCCL8, bIL‐12, and bLECT2 cannot be expressed likely due to strong metabolic burden and/or toxicity, leading to negative selection against stable expression in *E. coli*.

For in vivo evaluation, we used the *Apc^min/+^* mice, a well‐established *Apc* gene mutation mouse model that spontaneously develops CRC precursor lesions and then multiple tumors in the gastrointestinal tract. After antibiotic treatment of removing the gut microbiome, we gavaged *Apc^min/+^* mice every 2 weeks with our engineered EcN carrying CMCP (EcN‐I‐CMCP) or with a control strain carrying the same C6‐HSL‐inducible lysis system and mCherry reporter but lacking CMCP expression (EcN‐I‐vector). After confirming the successful colonization, lysis of EcN was induced by injecting C6‐HSL s.c. twice a week until 20 weeks (Figure 3c). Compared to the control group, EcN‐I‐bIL‐1 and EcN‐I‐bIL‐33 groups significantly maintained *Apc^min/+^* mice body weight, especially at 19–20 week (both group *p* < 0.01) (Figure 3d). After 20 weeks, all mice have their intestinal tracts harvested, tumor numbers and volumes were measured under a stereoscope. We found that in these *Apc^min/+^* mice, the number and volume of tumors were significantly lower in EcN‐I‐bIL‐1 and EcN‐I‐bIL‐33 groups relative to the controls (Figure 3e,f). In short, we found that CMCPs delivered by an engineered EcN influence tumor development in vivo, especially that bIL‐1/bIL‐33 that were depleted in CRC patients, carrying out significant anti‐tumor effects against tumor development. To gain deeper insight into CMCP expression in the engineered EcN strains, we performed additional in vitro and ex vivo analyses. We quantified the production of CMCPs in EcN‐I‐bIL‐1 and EcN‐I‐bIL‐33 strains using ELISA, which confirmed robust CMCP production in both strains (Figure S7a). Additionally, semi‐quantitative immunofluorescence staining on intestinal tissue sections ware performed to assess the expression of CMCPs in situ. This analysis further validated the presence and localization of CMCPs in the gut, supporting our in vivo observations (Figure S7b,c).

### 2.4 Histology and Immunostaining Reveal Immune Cell Recruitment by CMCPs

**FIGURE 4:**
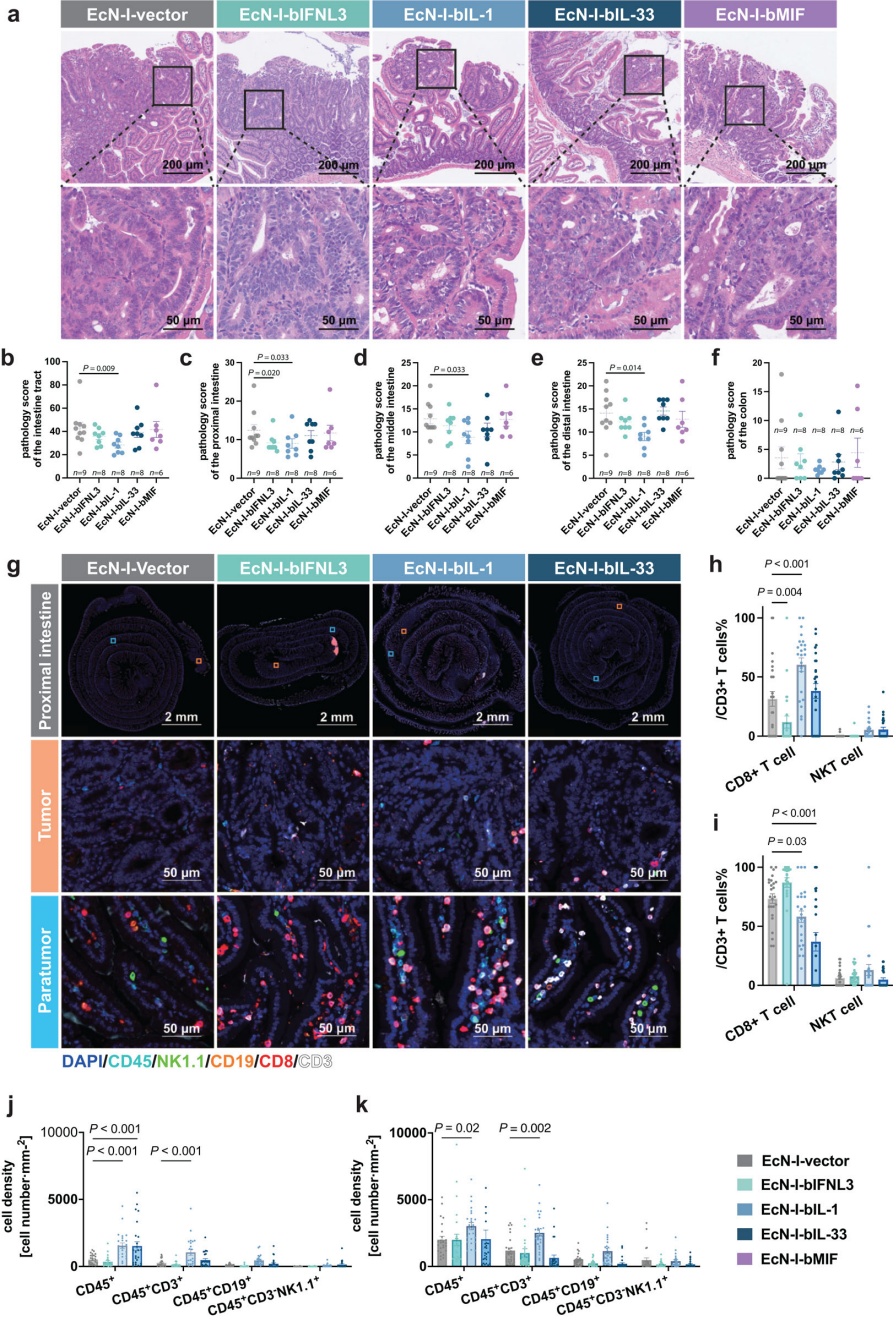
Immune cell recruitment by CMCPs in *Apc^min/+^* mouse intestine revealed by hematoxylin and eosin (H&E) staining and multiplex immunostaining (mIF). (a) The representative bright‐field, H&E staining of proximal intestinal sections in *Apc^min/+^* mice following treatment with engineered bacteria. Top row: 10× objective; bottom row: 40× objective. Scale bars: 200 µm (top row), 50 µm (bottom row). (b–f) Pathological scores of intestinal sections in *Apc^min/+^* mice following treatment with engineered bacteria. (b) whole intestinal tract, (c) proximal intestine, (d) middle intestine, (e) distal intestine, f. colon. Data are presented as the mean ± SEM. Significance is indicated directly in the figures with exact *p* values. *n* indicated the number of samples. (g) The representative mIF images of proximal intestinal sections in *Apc^min/+^* mice following treatment with engineered bacteria. Top row: 0.55× objective, proximal intestine; middle row: 40× objective, tumor area; bottom row: 40× objective, paratumor area. Scale bars: 2 µm (top row), 50 µm (middle and bottom row). (h,i) Percentages of CD8^+^ T cells and NKT cells within CD3^+^ T cells in tumor region (h) or in the paratumor region (i) following treatment with engineered bacteria. CD8^+^ T cells were identified as CD45^+^CD3^+^CD8^+^, ^and^ NKT cells were identified as CD45^+^CD3^+^NK1.1^+^. Quantification was performed on *n* = 5 mice per group, with 5 independent fields per mouse. Statistical analysis was performed by one‑way ANOVA followed by multiple comparisons using Dunnett's test. Data are presented as the mean ± SEM. Significance is indicated directly in the figures. (j,k) Densities of CD45^+^ cells, CD45^+^CD3^+^cells, CD45^+^CD19^+^cells and CD45^+^CD3^−^NK1.1^+^cells in tumor region (j) or in the paratumor region (h) following treatment with engineered bacteria. Quantification was performed on *n* = 5 mice per group, with 5 independent fields per mouse. Cells were counted and normalized to area. Statistical analysis was performed by one‑way ANOVA followed by multiple comparisons using Dunnett's test. Data are presented as the mean ± SEM. Significance is indicated directly in the figures.

We subsequently performed histological analysis and immunostaining of the intestinal tissues from *Apc^min/+^* mice inoculated with different CMCP‐delivering EcNs to further determine their effects. Hematoxylin‐eosin (HE) staining revealed that in the EcN‐I‐bIL1 group, a significantly lower level of inflammation and improved tissue integrity were observed in the small intestine parts compared to the control group (Figure 4a–f). Besides, we additionally found that the EcN‐I‐bIFNL3 group exhibited a better pathology score within the proximal intestine compared to the control (Figure 4c).

We also performed immunostaining against markers specific for B cells, T cells as well as NK cells, as the infiltration of the latter two cell types was mostly considered to be crucial for anti‐tumor immunity. To further investigate the spatial distribution and composition of immune cells within the tumor and surrounding tissues, tyramide signal amplification (TSA)‐based multiplex immunofluorescence (mIF) analysis was performed on EcN‐I‐bIFNL3, EcN‐I‐bIL1, and EcN‐I‐bIL33 proximal intestine samples (Figure 4g–k). This analysis enabled the quantification of distinct immune cell subsets, including CD8^+^ T cells (CD45^+^ CD3^+^ CD8^+^), NKT cells (CD45^+^ CD3^+^ NK1.1^+^), B cells (CD45^+^ CD19^+^), and natural killer (NK) cells (CD45^+^ CD3^−^ NK1.1^+^). We compared the abundance of those immune cells both within the tumor core and around the peritumoral area, found that in the EcN‐I‐bIL‐1 and EcN‐I‐bIL‐33 groups, there was a significantly higher number of immune cell (CD45^+^ cells) within the tumor area compared to the control group (Figure 4j). In particular, in the EcN‐I‐bIL‐1 group, significantly higher infiltration of T cells (CD45^+^CD3^+^ cells) was observed within the tumor, especially concentrated at the tumor margin (Figure 4g,j). Moreover, the pronounced tumor‐eliminating effect of EcN‐I‐bIL‐1 group can be attributed to the significant increase in CD8^+^ T cells (Figure 4h). Compared to EcN‐I‐bIL‐33, EcN‐I‐bIL1 group tended to concentrate CD8^+^ T cells around the tumor area (Figure 4h,i). The EcN‐I‐bIL‐33 group additionally showed higher B‐cell infiltration (Figure S8). In short, we found that the selected CMCPs inhibit tumor growth in *Apc^min/+^* mice, significantly elevated the abundance of anti‐tumor immune cells, particularly T cells in the tumor, ostensibly contributing to the anti‐tumor immune responses.

### 2.5 Immunomodulatory Functions of CMCPs Revealed by RNA‐seq

**FIGURE 5:**
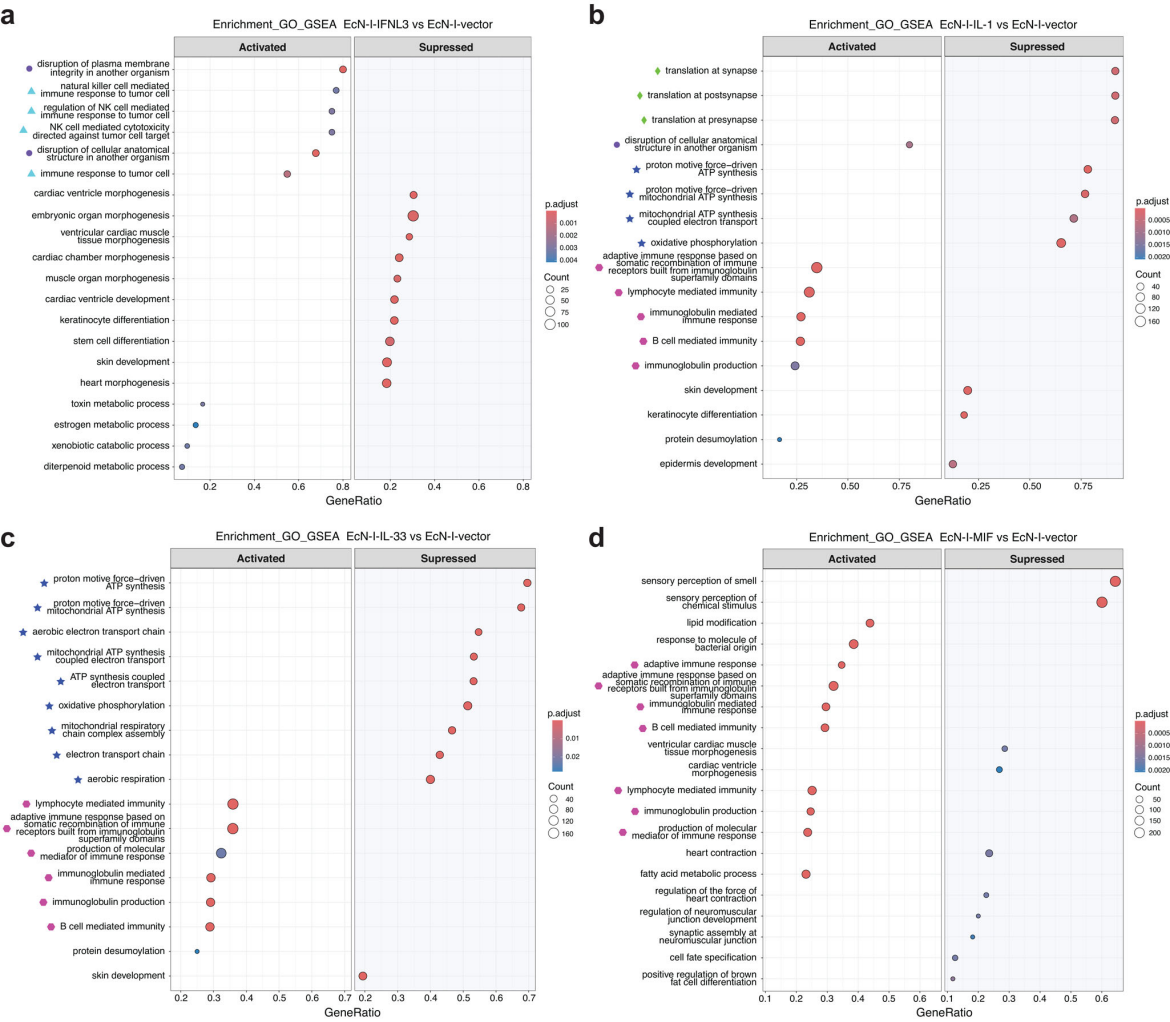
GSEA analysis of the intestinal transcriptome in *Apc^min/+^* mice following treatment with engineered bacteria. Top 10 terms are shown for each comparison. The size of the bubble represents the strength of the correlation, and the color of the bubble represents the adjusted *p*‐value (*padj*). Blue represents the pathway is down‐regulated, red represents the pathway is up‐regulated. Shapes to the left of pathway names indicate pathway groupings; identical shapes denote pathways that belong to similar functional categories. (a) EcN‐I‐bIFNL3 versus Ctl, (b) EcN‐I‐bIL‐1 versus Ctl, (c) EcN‐I‐bIL‐33 versus Ctl, and (d) EcN‐I‐bMIF versus Ctl. Significance was determined using the Benjamini–Hochberg procedure to control the false discovery rate (FDR), and pathways with *padj* < 0.05 were considered significant.

To elucidate the mechanism underlying the different effects of those CMCPs in colorectal cancer development, we performed RNA‐seq analysis of duodenum tissues across various EcN‐I‐CMCP treatment groups. In each group, we discovered between 217 and 1,046 upregulated differentially expressed genes (DEGs), and between 340 and 736 downregulated DEGs, compared to the control group (Figure S9a–d). A consequent Gene Set Enrichment Analysis (GSEA) identified biological processes mostly affected by our CMCPs, including translational effects, influencing pathways associated with both anti‐ and pro‐tumor activities, immune modulation, energy metabolism, and cell structure maintenance (Figure 5a–d).

Notably, in the EcN‐I‐bIL‐1 group that had the most significant anti‐tumor effect, pathways related to adaptive immune responses, particularly those involving B and T cell‐related, were upregulated (Figure 4), ostensibly aiding tumor elimination. Conversely, the most significantly downregulated pathways include those related to energy metabolism and synaptic translation, which may contribute to suppressing tumor‐associated energy metabolism and intestinal neurophysiological signaling (Figure 5b). The EcN‐I‐bIL‐33 group exhibited a similar transcriptional footprint to EcN‐I‐bIL‐1. Although EcN‑I‑bIFNL3 and EcN‑I‑bMIF did not produce significant reductions in tumor number or volume under the tested conditions, we nonetheless found that they affected gene expression in several pathways related to immune response to tumor (Figure 5c). In the EcN‐I‐bIFNL3 group, NK cell‐related immune response pathways were upregulated, indicating that bIFNL3 still induces a tumor‐targeted response (Figure 5a). In the EcN‐I‐bMIF group, upregulation of adaptive immune pathways was also found (Figure 5d). Thus, RNA‐seq analysis revealed significant impacts of CMCPs on anti‐tumor immunity, especially T cells that agree with histological and immunological findings.

### 2.6 Heterologously Expressed CMCPs Incite Immune Cell Responses In Vitro

**FIGURE 6:**
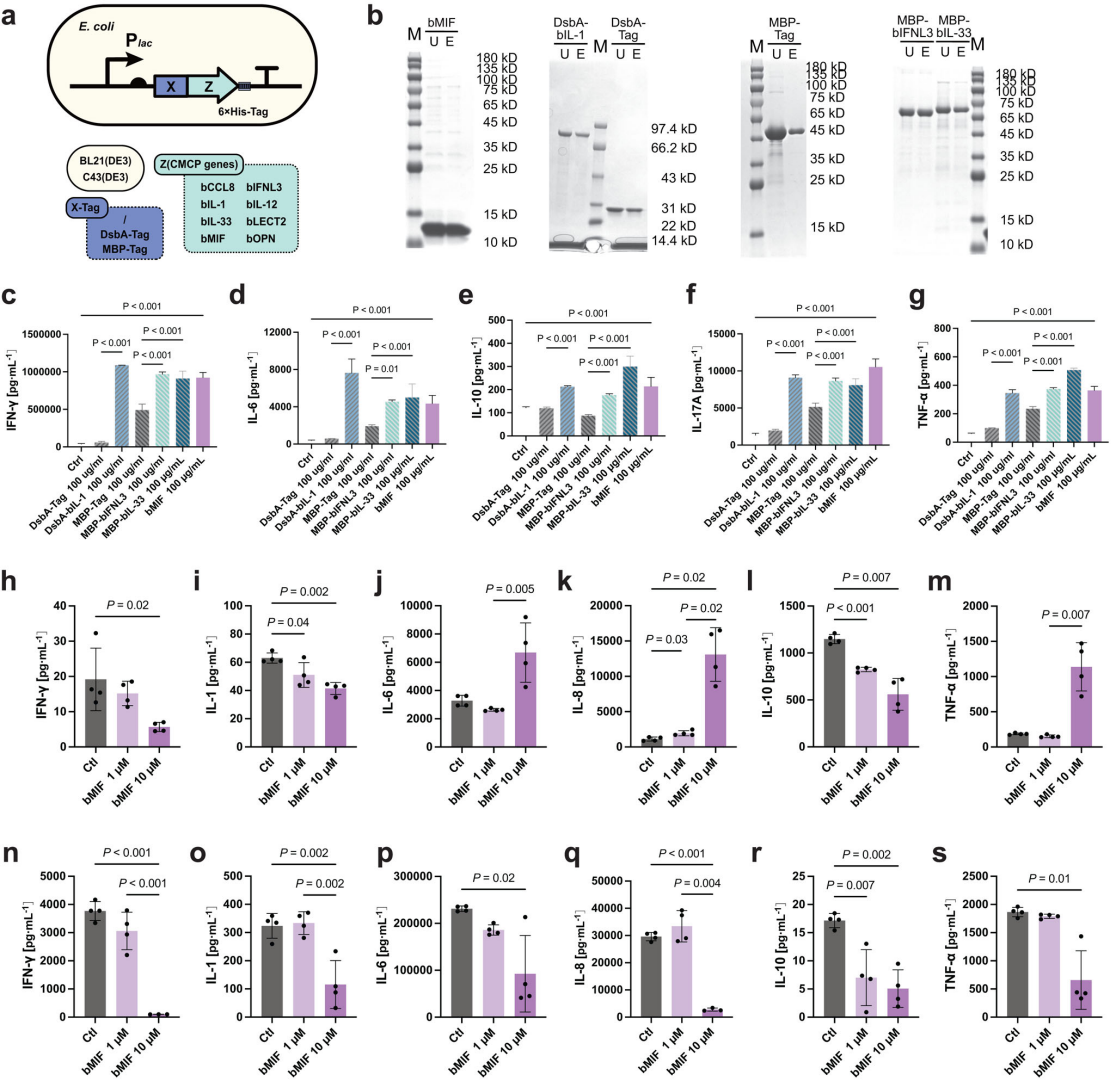
Recombinant CMCPs activate T cells, dendritic cells, and macrophages in vitro. (a) Schematic diagram of pET‐protein‐purification system in *E. coli*. (b) Protein SDS‐PAGE of bIFNL3, bIL‐1, bIL‐33, bMIF, bOPN, and their corresponding tags concentration and post‐endotoxin removal. (c–g) The concentration of cytokines (pg·mL^−1^) in different treatment groups was quantified using enzyme‐linked immunosorbent assay (ELISA) while CMCPs stimulating mouse splenic T cells. Statistical analysis was performed by one‑way ANOVA (or Kruskal–Wallis depend on the data type) followed by multiple comparisons using Tukey's test. Data are presented as the mean ± SEM, *n* = 3. Significance is indicated directly in the figures. (c) IFN‐γ, (d) IL‐6, (e) IL‐10, (f) IL‐17A, (g) TNF‐α. (h–m) The concentration of cytokines (pg·mL^−1^) in different treatment groups was quantified using enzyme‐linked immunosorbent assay (ELISA) while bMIF stimulating mouse bone marrow‐derived dendritic cells. Statistical analysis was performed by one‑way ANOVA (or Kruskal–Wallis depend on the data type) followed by multiple comparisons using Dunnett's test. Data are presented as the mean ± SEM, *n* = 4. Significance is indicated directly in the figures. (h) IFN‐γ, (i) IL‐1, (j) IL‐6, (k) IL‐8, (l) IL‐10, (m) TNF‐α. (n–s) The concentration of cytokines (pg·mL^−1^) in different treatment groups, quantified using enzyme‐linked immunosorbent assay (ELISA) while bMIF stimulating mouse bone marrow‐derived macrophages. Statistical analysis was performed by one‑way ANOVA (or Kruskal–Wallis depend on the data type) followed by multiple comparisons using Dunnett's test. Data are presented as the mean ± SEM, *n* = 4. Significance is indicated directly in the figures. (n) IFN‐γ, (o) IL‐1, (p) IL‐6, (q) IL‐8, (r) IL‐10, (s) TNF‐α.

We next expressed the differentially enriched CMCPs in *E. coli* BL21(DE3) and C43(DE3) systems and purified the products for further in vitro functional studies, in order to directly examine the immune responses and anti‐tumor effects of CMCPs and remove potential confounding factors in vivo. Coding sequences were codon optimized on a pET‐28a plasmid, a commonly used IPTG‐concentration‐regulated plasmid for better *E. coli* expression (Figure 6a). In combination of several purification, expression, and secretion tags (6×His, DsbA, Ffu209/217/312, MBP, OmpA, OmpF, OsmY, SUMO, TorA, and YebF), we eventually obtained soluble forms of bIFNL3, bIL‐1, bIL‐33, and bMIF, with molecular size agreeing with expectations in SDS‐PAGE and protein sequence confirmed by mass spectrometry (Figure 6b). Similar to the case in EcN, bCCL8, bIL‐12, and bLECT2 cannot be expressed in BL21(DE3) or C43(DE3) strains, likely due to misfolding, toxicity or burden on *E. coli*.

Using these purified CMCPs, we examined their direct effects on mouse immune cells, including splenic T cells activated by CD3/CD8, bone marrow‐derived macrophages (BMDMs), and bone marrow‐derived dendritic cells (BMDCs). Cytokine secretion (IFN‑γ, IL‑6, IL‑10, IL‑17A, and TNF‑α) was quantified by ELISA. We found all of the tested CMCPs exert the most significant impact on T cells, as treatment with bIFNL3, bIL‐1, bIL‐33, and bMIF led to significantly increased secretion of IFN‐γ, IL‐6, IL‐10, IL‐17A, and TNF‐α compared to the tag‐only control group (Figure 6c–g). Moreover, we observed similar effects in human peripheral blood mononuclear cells (hPBMCs), where CMCP treatment also induced significant cytokine secretion (Figure S10a–e). These findings suggest that CMCPs can activate immune responses in both mouse and human immune cells. In mouse myeloid cells, we additionally found that bMIF enhanced activation‑associated responses in BMDMs (Figure 6h–m) but inhibited BMDCs (Figure 6n–s). Together, these in vitro results indicate that the expressed CMCPs directly modulate mouse immune‑cell functions, particularly T‑cell responses, making it unlikely that the in vivo effects we observed are solely indirect artifacts.

To further investigate the mechanistic basis of CMCP‐mediated immunomodulation, particularly receptor recognition, we performed additional receptor‐binding and functional validation experiments. Specifically, we conducted Bio‐Layer Interferometry (BLI) assays and utilized a heterologous expression system in HEK293T cells expressing the corresponding canonical cytokine receptors, testing the receptor–ligand pairs bIL1–IL1R1 and bIL33–IL1RL1 (Figure S11a–g). Under the tested conditions, however, we did not detect measurable direct binding between CMCPs and these classical cytokine receptors.

## 3 Discussion

In this study, we characterized a new subset of bacterial proteins, namely cytokine motif‐containing, immunomodulatory bacterial proteins (CMCPs), and investigated their potential roles in modulating the gut microenvironment and host anti‐tumor immunity. We demonstrate that bacteria can also encode molecules similar to mammalian cytokines, capable of modulating host immunity and gut homeostasis. Using the engineered probiotic *Escherichia coli* Nissle 1917 (EcN), we show that specific CMCPs promote immune‑cell proliferation and differentiation, thereby enhancing immune‐mediated tumor control in vivo. Together, these results support that CMCPs exert multifaceted effects on host immunity and tumor biology, demonstrating their potential utility in microbiome‑based cancer immunotherapies.

Despite their limited size, peptides and small proteins are known to play significant roles in diverse biological processes. For example, they serve as key components in innate immunity (e.g. defensins [39, 40] and cytokines [41, 42]) and as regulators of metabolism and neural functions (e.g., peptide hormones [43, 44, 45]). Historically, immunomodulatory studies on gut microbiome have focused on small molecules—metabolites (e.g., short‐chain fatty acids and tryptophan derivatives) and signaling lipids, owing to their rapid diffusion and broad systemic effects [7, 8]. However, emerging evidence indicates that many proteins encoded by small ORFs in bacteria can also exert highly specific activities, such as anti‐microbial peptides (AMPs) [14, 15, 16]. Among which, many exert potent immunomodulatory effects (e.g., Nisin) [46, 47]. Our results on CMCPs extend this emerging concept, suggesting that a substantial reservoir of bacterial proteins may contribute to immune regulation, barrier function, and disease modulation

As an illustrative example, bMIF displayed context‐dependent immunomodulatory activities across multiple immune cell types. Although bMIF was enriched in healthy individuals (Figure 2d), suggesting an association with immune homeostasis, our in vitro assays showed that it suppresses BMDC activation under simplified experimental conditions (Figure 6n–s). These findings are not interpreted as contradictory. In vivo, immune responses arise from complex interactions among diverse immune populations, cytokine networks, and tissue‐specific cues. While bMIF dampens dendritic cell activation in isolation, it may simultaneously activate T cells and macrophages, with the net immunological effect likely shaped by the balance of these cell‐type‐specific activities within the physiological environment.

Importantly, suppression of dendritic cell overactivation does not necessarily imply impaired antitumor immunity. In certain contexts, limiting excessive DC activation may help prevent chronic inflammation or immune dysregulation, thereby supporting immune homeostasis. This bidirectional regulatory pattern is reminiscent of the pleiotropic roles described for mammalian MIF, whose functions vary depending on cellular context and microenvironment [48, 49, 50]. Together, these findings suggest that bMIF acts not as a unidirectional immunosuppressive factor but as a context‐dependent regulator of immune balance, highlighting the importance of evaluating CMCP functions within integrated in vivo systems.

Functionally, CMCPs can be conceptually compared with other classes of microbial immunomodulatory proteins, including bacterial superantigens and viral cytokine mimics (virokines), while remaining clearly distinct from these other classes. Superantigens typically trigger extensive, non‐specific T cell activation through direct crosslinking of MHC class II molecules and T cell receptors, often leading to pathological immune activation [51, 52]. In contrast, CMCPs do not induce such overt polyclonal responses but instead elicit more selective and context‐dependent immune modulation. Likewise, whereas many virokines exhibit strong structural and functional mimicry of host cytokines and engage canonical cytokine receptors, our binding and receptor‐expression assays indicate that CMCPs do not measurably interact with classical cytokine receptors. (Figure S11) Considering that the cross‐reactivities are extremely common between cytokine/chemokine and the receptors, it's likely that the canonical receptors known to human cytokines are not used by those bacterial proteins, but instead other receptors are involved, and resolving the exact targets requires further dedicated studies. These observations suggest that CMCPs may represent a distinct mode of microbial immune modulation that does not rely on direct molecular mimicry, but rather on alternative or indirect pathways of immune engagement.

From an evolutionary standpoint, the limited sequence and structural similarity between CMCPs and host cytokines raises the possibility that their immunomodulatory capacity did not arise through direct co‐evolution with host immune receptors. Instead, CMCPs may reflect a form of functional convergence, whereby bacterial proteins acquire the ability to influence host immunity without preserving overt homology to host signaling molecules. Such a strategy may enable flexible immune modulation while avoiding strong selective pressure from host immune surveillance mechanisms. On top of that, from the perspective of the bacteria themselves, it is worth considering whether CMCPs serve as accidental evolutionary byproducts or if they confer specific advantages—such as modulating host immune responses to facilitate colonization, persistence, or even pathogenesis. This speculative hypothesis highlights the potential evolutionary advantages of CMCPs in microbial‐host interactions, where subtle immune modulation could play a critical role in microbial survival and host adaptation. Although these possibilities remain to be experimentally tested, incorporating them into the discussion provides a broader evolutionary context for understanding CMCPs and their role in microbial immunomodulation.

With the identification of CMCPs, which naturally encode in the gut microbiome, and their delivery via engineered probiotics (e.g., EcN) directly to the tumor microenvironment, we achieved tumor‑restricted release that minimizes damage to normal tissues. We speculate that combining bacterially derived CMCPs with established immunotherapies may further potentiate anti‐tumor immune responses and expand the scope of combinatorial treatment strategies. Additionally, integrating adoptive cell therapies, such as CAR‐T or CAR‐NK, with CMCPs may help overcome challenges such as target selection and poor immune cell, especially effector cell trafficking and infiltration in solid tumors. Engineered bacteria, with their potential as ‘cell factories,’ can continuously or systematically synthesize and deliver short half‐life therapeutics, offering new possibilities for clinical application. Nonetheless, this approach still requires a more precise systematic evaluation of synergistic efficacy, safety (including potential systemic inflammation, vector immunogenicity, etc.), and optimized delivery and dosing regimens. A promising direction for future studies would be to investigate whether the abundance of CMCPs in the tumor microenvironment correlates with clinical parameters such as tumor stage, grade, or patient survival, which could provide further insights into their potential as biomarkers for treatment stratification.

In terms of cancer therapy, conventional approaches such as surgery, radiotherapy, and chemotherapy are increasingly complemented by immunotherapies and targeted therapies, yet issues of safety and efficacy remain [53, 54]. One major type of immune therapy, immune checkpoint inhibitors (ICIs) has demonstrated promising results in multiple malignancies. However, their efficacy is frequently hindered by the heterogeneous and immunosuppressive tumor microenvironment [55, 56]. In contrast, cytokine‐based therapies have been limited in clinical application due to risks like immune storms and insufficient in vivo bioactivity [57, 58]. With the identification of CMCPs, which naturally encode in the gut microbiome, and their delivery via engineered probiotics (e.g., EcN) directly to the tumor microenvironment, we achieved tumor‑restricted release that minimizes damage to normal tissues. We speculate that combining bacterially derived CMCPs with established immunotherapies may further potentiate anti‐tumor immune responses and expand the scope of combinatorial treatment strategies. Additionally, integrating adoptive cell therapies, such as CAR‐T or CAR‐NK, with CMCPs may help overcome challenges such as target selection and poor immune cell, especially effector cell, trafficking and infiltration in solid tumors [59, 60, 61].

Engineered bacteria, with their potential as “cell factories”, can continuously or systematically synthesize and deliver short half‐life therapeutics, offering new possibilities for clinical application [62, 63]. Nonetheless, this approach still requires a more precise systematic evaluation of synergistic efficacy, safety—particularly addressing concerns such as potential systemic inflammation, vector immunogenicity, and off‐target effects—and optimized delivery and dosing regimens. A promising direction for future studies would be to investigate whether the abundance of CMCPs in the tumor microenvironment correlates with clinical parameters such as tumor stage, grade, or patient survival, which could provide further insights into their potential as biomarkers for treatment stratification.

Our findings could still benefit from further in‐depth examinations on the CMCPs, from both mechanistic and applicational points of view. Although these peptides harbor cytokine‐associated motifs and elicit robust immune responses, the receptors they engage and the signaling circuits they initiate are unknown, raising the possibility that they either co‐opt canonical cytokine pathways or operate through unrecognized mechanisms. A major limitation lies in the quantitative assessment of CMCP release in vivo, where technical constraints impede direct comparison with host cytokines and obscure their physiological relevance. Future studies will be critical to fully elucidate CMCP function, including improving quantification methods, exploring alternative delivery strategies (e.g., nanoparticles or mRNA‐based platforms), and expanding mechanistic analyses. Addressing these gaps will require systematic screening of additional CMCPs for immunological activity, rigorous validation through receptor‐blocking and functional assays, and high‐resolution structural interrogation by nuclear magnetic resonance (NMR) or cryogenic electron microscopy (cryo‐EM) to resolve structure–function relationships. With more thorough investigation, integrating abundance, molecular, structural, and functional insights of CMCPs with clinical parameters such as tumor stage, grade, or patient survival may clarify their relevance in disease progression and therapeutic response, particularly in the context of ICI‐based regimens. The establishment of a comprehensive, publicly accessible database of CMCPs linking molecular profiles to functional and clinical annotations would provide a foundation for mechanistic discovery and accelerate the development of precision immunomodulatory strategies.

In summary, our investigations on CMCPs not only broaden current understanding of gut‐microbiota‐host interaction mechanisms but also offer novel molecular resources and engineering strategies for tumor immunotherapy. Future research should further delineate CMCP mechanisms across diverse tumor microenvironments, and by integrating multi‐omics technologies with engineered bacterial delivery systems to enable mechanistic insight, patient stratification, and rational design of personalized, precision therapeutics.

## 4 Experimental Section

### 4.1 Public Data Collection and Processing

The list of cytokines was obtained from the ImmPort Cytokine Registry (https://www.immport.org/resources/cytokineregistry) after removing entries containing the keyword “receptor”. Entrez Gene official names and Entrez Gene IDs (human) were used as queries for sequence retrieval and HMM searches. These terms were searched in Pfam and NCBI to identify existing HMM models and to retrieve sequences for model construction, respectively. Bacterial genomes were downloaded from the NCBI RefSeq prokaryotic collection (Bacteria taxonomy) [64]. Coding sequences (CDS) and exons were extracted using custom scripts that referenced the corresponding GFF annotation files. CRC cohort metagenomes were obtained from publicly available studies. The raw data for metagenomes used in this study are publicly available in NCBI Sequence Read Archive (SRA) under the BioProject IDs PRJDB4176 [65], PRJEB10878 [66], PRJEB12449 [67], PRJEB27928 [68], PRJEB6070 [69], PRJEB7774 [70], PRJNA397219 [71], and PRJNA447983 [72]. This study included samples from colorectal cancer (CRC) patients and healthy controls; samples from patients with adenomas were excluded. Metagenomic reads were downloaded and processed for quality control. Kneaddata (v0.7.6) was used for sequence quality control [73]. Trimmomatic (v0.39) was applied to trim adapters, and low‑quality reads and host‑derived reads were also removed [74]. Megahit (v1.2.9) was used for metagenome assembly [75]. Getorf (EMBOSS v6.6.0.0) was used to extract open reading frames with the parameters “‐find 0‐table 11‐minsize 150”, retaining sequences ≥ 150 nt (≥ 50 amino acids) [76]. The proteomic data used in this study are publicly available in PRoteomics IDEntifications Database (PRIDE) under the accession numbers PXD000114 [77], PXD003907 [78], PXD005780 [79], and PXD008870 [80].

### 4.2 HMM Building and Mining

Except for HMMs obtained from public databases, remaining models were constructed with HMM build (HMMER v3.2.1) [81]. Input sequences for model construction were first aligned using MUSCLE (v3.8.1551) [82]. Model performance was evaluated by 10‑fold cross‑validation. Sequences were evenly and randomly partitioned into *K* = 10 subsets; for each fold k (k = 1–10), the k‑th subset served as the validation (test) set and the remaining *K*−1 = 9 subsets as the training set. For each fold, a multiple sequence alignment of the training set was used to build an HMM using HMM build, and the resulting model was applied to the test set using HMM search. Predictions were recorded and computed for fold accuracy. Fold accuracy was defined as the number of correctly predicted samples divided by the total number of samples in that test set. The overall cross‑validated accuracy reported is the mean accuracy across the 10 folds.

(1) $$A_{i}=\frac{C_{i}}{T_{i}}$$

Let *C_i_* denote the number of correctly predicted samples in the *i*‑th fold and *T_i_* the total number of samples in that fold. The accuracy of fold *i* is therefore *A_i_*. After completing training and validation for all *K* folds, the overall cross‑validated accuracy was summarized as the mean fold accuracy:

(2) $$A_{avg}=\frac{1}{K}\sum_{i=1}^{K}A_{i}$$

After computing *A_avg_*, putative confounding sequences—for example, instances in which the same annotation symbol maps to different proteins—that were suspected of contributing to low model accuracy were iteratively removed. Model construction and cross‑validation were then repeated until *A_avg_* exceeded 50%.

CMCP mining in both bacterial genomes and CRC metagenome was performed using the HMM search module with default parameters. Candidate hits with reported *E‑values* < 0.01 were retained, and the corresponding sequences and associated metadata were extracted for downstream analyses. Redundant sequences were removed with CD‑HIT (v4.7) using the parameters ‘‐c 0.95‐n 5‐G 0‐aS 0.9‐g 1‐d 0‐T 20‐M 0’ [83, 84].

### 4.3 Phylogenic Tree Building

Sequences of CMCPs mined from bacteria genomes, vertebrate (mammals and others) genomes and invertebrate genomes were redundancy eliminated with CD‐HIT, aligned with Clustal Omga (v1.2.4) [85, 86] using default parameters, and constructed phylogenic trees with fast tree (v2.2) [87, 88]. Trees were visualized using the iTOL (Interactive Tree Of Life, v7.2.2) web tool [89].

### 4.4 Relative Abundance Calculation and Meta‐Analysis

Sequencing reads were aligned to contigs containing the identified CMCPs using Bowtie2(v 1.2.2) [90], and SAMtools (v1.9) [91] was used to convert, sort, and index alignment files for mapping‑quality assessment. Finally, Linux shell and Python 3 scripts were used to count reads mapping to the cytokine homologs in each metagenomic sample and to compute their relative abundances. For each cohort, CMCP abundance tables were analyzed in R using the DESeq package (v1.46.0) together with GenomicFeatures (v1.58.0) to test and show for differential abundance between colorectal cancer (CRC) patients and healthy controls. Log‑fold changes (LFC) and associated *adjusted p‐values (padj)* were extracted from each cohort‐level analysis. Volcano plots were generated with the ggplot2 package (v3.5.1), highlighting features with |LFC| > 2 and *padj* < 0.05.

Cohort‑level summary statistics were then combined across the eight cohorts using METAL (v generic‑metal‑2011‑03‑25) [92] to identify cytokine homologs that were consistently up‑ or down‑regulated in CRC patients relative to controls. Identified candidates were interpreted as putative promoters or inhibitors of CRC based on the direction and consistency of effect across cohorts.

### 4.5 Protein Sequence Alignment, Structure Prediction, and Alignment

Sequence similarities were aligned using BLASTp (v2.10.1) with default parameters [93]. Three‑dimensional structures of candidate CMCPs were predicted using AlphaFold 3 (DeepMind and Isomorphic Lab) with default parameters [94]. Predicted models were aligned with the corresponding human cytokine structures retrieved from the AlphaFold Protein Structure Database [95, 96]. Structural alignments were performed with PyMOL (v3.1.0) and complementary alignment software, applying the “align” command for globally similar molecules, “cealign” for cases of low sequence similarity, and “US‑align” for pairs exhibiting substantial structural divergence [97]. Alignment quality was assessed using standard metrics (e.g., root‑mean‑square deviation, RMSD) and AlphaFold structure confidence scores (e.g., pLDDT/PAE), which were recorded for each structure.

### 4.6 Strains and Plasmids

All plasmids were assembled using the Gibson assembly method. [98]. For recombinant protein expression, *Escherichia coli* (*E. coli*) BL21(DE3) and C43(DE3) (C504, Vazyme Biotech Co., Ltd.) carrying pET28a‐based expression constructs (Saisofi Biotechnology Co., Ltd.) were used. Candidate coding sequences (*bccl8*, *bifnl3*, *bil1*, *bil12*, *bil33*, *blect2*, *bmif*) were synthesized and cloned into pET28a under the P*_lac_* promoter with a C‑terminal 6×His tag. To improve solubility, *bifnl3* and *bil33* constructs included an N‑terminal MBP tag, and *bil1* included an N‑terminal DsbA tag. All coding sequences were codon‑optimized for better expression in *E. coli*. Correct assemblies were confirmed by colony PCR and Sanger sequencing. Plasmids were introduced into chemically competent cells by heat shock. Cultures were grown in LB medium at 37°C with shaking at 180 rpm, supplemented with kanamycin (30 µg·mL^−1^).

To construct engineered strains intended for tumor colonization, *Escherichia coli* Nissle 1917 (EcN) from our laboratory collection was used. The pCRCTS1 backbone carrying a C6‑HSL‑inducible lysis cassette was kindly provided by Jin'e Li Lab (IMCAS) [38]. Five candidate coding sequences were cloned downstream of the P*_vioA532_* promoter, positioned between *X174E* and *mCherry*. Constructs were verified by Sanger sequencing and subsequently introduced into EcN by electroporation. Engineered EcN strains were maintained in LB medium at 37°C with shaking at 180 rpm, supplemented with chloramphenicol (34 µg·mL^−1^).

### 4.7 Protein Purification

Strains were cultured in LB medium at 37°C with shaking at 180 rpm, supplemented with kanamycin (30 µg·mL^−1^), until an OD_600_ of ∼0.6 was reached. Cultures were then induced with isopropyl‐β‐D‐thiogalactopyranoside (IPTG) at varying concentrations and incubated at different temperatures for specified durations. The optimized expression and purification conditions used in this study are summarized in Table 1.

**TABLE 1:** Protein expression and purification conditions.

Protein	Culture strain and condition	Cell lysis method
MBP‐bCCL8	BL21(DE3) 28°C 180 rpm 0.5 mM IPTG 16 h	supersonification
MBP‐bIFNL3	BL21(DE3) 28°C 180 rpm 0.5 mM IPTG 4 h	supersonification
DsbA‐bIL1	BL21(DE3) 28°C 180 rpm 0.1 mM IPTG 4 h	supersonification
MBP‐bIL33	BL21(DE3) 28°C 180 rpm 1 mM IPTG 8 h	supersonification
bMIF	C43(DE3) 37°C 180 rpm 0.1 mM IPTG 16 h	lysis buffer
bOPN	BL21(DE3) 37°C 180 rpm 0.1 mM IPTG 16 h	supersonification
DsbA‐Tag	BL21(DE3) 28°C 180 rpm 0.1 mM IPTG 4 h	supersonification
MBP‐Tag	BL21(DE3) 28°C 180 rpm 0.1 mM IPTG 4 h	supersonification

Cells were harvested by centrifugation (12 000×*g*, 1 min, 4°C) and washed with PBS. Cell pellets were resuspended in PBS supplemented with protease inhibitor (P1030; Beyotime Biotechnology Co., Ltd.) and lysed on ice either by sonication (90 W, 2 s‐on/3 s‐off cycles, total sonication time 30 min) or by using cell lysis buffer for Western blot and immunoprecipitation (P0013; Beyotime Biotechnology Co., Ltd.). Lysates were clarified by centrifugation (12 000×*g*, 1 min, 4°C), and the supernatant was filtered through a 0.22 µm syringe filter prior to affinity purification.

Depending on the fusion tag, clarified lysates were applied to prepacked Ni‑NTA resin (Sangon Biotech (Shanghai) Co., Ltd.) for His‑tagged proteins or to Dextrin Resin (Beijing Solarbio Science & Technology Co., Ltd.) for MBP‑tagged proteins. Following elution according to the manufacturer's instructions, proteins were analyzed by SDS–PAGE and identified by mass spectrometry. Buffer exchange into PBS and simultaneous concentration were performed using ultrafiltration units (Amicon Ultra‑15 Centrifugal Filter Units). Protein concentration was determined with a BCA Protein Assay Kit (P0011; Beyotime Biotechnology Co., Ltd.). Purified proteins were aliquoted and stored at −80°C.

### 4.8 Quantitative Analysis of Fluorescence Intensity

Cultures were grown overnight in LB medium at 37°C with shaking at 180 rpm in the dark, supplemented with chloramphenicol (34 µg·mL^−1^) and C6‑HSL (1 µM). RFP fluorescence was measured using a Synergy H4 multi‑mode plate reader (BioTek, Winooski, VT, USA). Aliquots collected at the indicated time points were transferred to 96‑well half‑area black plates (Corning Incorporated) for measurement. Fluorescence was recorded in relative fluorescence units (RFU). Background fluorescence was subtracted from each measurement. Prior to reading, plates were shaken in the plate reader orbitally for 15 s at medium speed; fluorescence was recorded with the focal height set to 1.5 mm with no instrument gain. Excitation and emission wavelengths for mCherry were 587 and 610 nm, respectively. Instrument settings were held constant across conditions. OD_600_ was recorded concurrently.

Quantitative Analysis of His‐tagged CMCP: Cultures were grown overnight in LB medium at 37°C with shaking at 180 rpm in the dark, supplemented with chloramphenicol (34 µg·mL^−1^) and C6‑HSL (1 µM). Cells were harvested by centrifugation (4000×*g*, 5 min) and resuspended in fresh LB medium supplemented with chloramphenicol (34 µg·mL^−1^) and C6‑HSL (1 µM) to an optical density at OD_600_ of 1.0. The cultures were then incubated for an additional 24 h under the same conditions. Following incubation, cultures were centrifuged at 12 000×*g* for 2 min to collect the supernatant. The concentration of His‐tagged CMCP in the supernatant was quantified using a His‐tag ELISA kit (HBDY‐91909O1, Beijing Huadeboyi Biotechnology Co., Ltd.).

### 4.9 Luciferase Reporter Assay

HEK293T cells stably expressing a luciferase reporter were seeded in 96‐well plates and co‐transfected with pCDH‐human IL1R1 reporter plasmids using polyethyleneimine (PEI). Twenty‐four hours post‐transfection, the culture medium was removed, and the cells were washed twice with DMEM. Subsequently, 100 µL of various concentrations of rhIL‐1β (CG93, Novoprotein), rhIL‐33 (C091, Novoprotein), DsbA‐Tag, DsbA‐IL1, MBP‐Tag, MBP‐IL33, rhIL‐1, or rhIL‐33 were added to each well, followed by a 24‐h incubation. After discarding the supernatant, 30 µL of reporter lysis buffer was added to each well, and cells were lysed on ice for 1 h. Following full lysis, the lysates were centrifuged at 10 000–15 000×*g* for 3–5 min at 4°C. The resulting supernatants were collected, and firefly luciferase activity was measured using a Firefly Luciferase Reporter Gene Assay Kit (RG007, Beyotime Biotechnology Co., Ltd.) according to the manufacturer's instructions on a luminometer.

### 4.10 Mouse Cell Culture

All cell cultures were maintained at 37°C in a humidified incubator with 5% CO_2_. Mouse splenic cells were cultured in RPMI 1640 (Gibco, Thermo Fisher Scientific) supplemented with 10% fetal bovine serum (FBS; ATCC) and 1% Pen/Strep (100 U·mL^−1^ penicillin and 100 µg·mL^−1^ streptomycin). GM‑CSF‑derived bone marrow‑derived dendritic cells (GM‑BMDCs) and M‑CSF‑derived bone marrow‑derived macrophages (M‑BMDMs) were maintained in RPMI‐10 medium (RPMI 1640 containing 10% fetal bovine serum, 1% Pen/Strep, 10 mM HEPES, 1% sodium pyruvate, 1% β‐mercaptoethanol (β‐ME), and 1% L‐glutamine).

### 4.11 Mouse Spleen Cell Generation

Mice were euthanized in accordance with institutional guidelines, and spleens were aseptically excised and transferred to ∼2 mL RPMI 1640 supplemented with 10% FBS and 1% Pen/Strep. Spleens were minced and mechanically dissociated by pressing through a 70 µm cell strainer using a sterile plunger. The resulting cell suspension was collected in a 15 mL tube; two volumes of RBC lysis buffer (R1010, Beijing Solarbio Science & Technology Co., Ltd.) relative to the suspension volume were added, mixed gently, and incubated for 10 min at room temperature. The mixture was then centrifuged at 1000 rpm for 6 min at room temperature, the supernatant was discarded, and the pellet was resuspended in 2 mL culture medium. The suspension was passed through a flow cytometry tube with a filter cap; after transfer, the tubes were centrifuged again (1000 rpm, 6 min) and the supernatant removed. Cells were resuspended, counted, and collected for subsequent culture or differentiation. For cell differentiation, anti‐CD3 (2 µg·mL^−1^, 108567‐T08, Sino Biological China Inc.) and anti‐CD8 (2 µg·mL^−1^, 50389‐R208, Sino Biological China Inc.) antibodies were added to stimulate T cell differentiation, and LPS (50 µg·mL^−1^, Beijing Solarbio Science & Technology Co., Ltd) was used for B cell.

### 4.12 Mouse BMDM and BMDC Generation

Mice were euthanized in accordance with institutional guidelines. The femurs and tibias were harvested, and the bone marrow was aseptically collected. Mice were sacrificed, and bone marrow was harvested from femurs and tibias. Bone marrow cells were resuspended in RBC lysis buffer and treated according to the manufacturer's instructions to remove erythrocytes; cells were then washed and pelleted. Cells were counted and resuspended in RPMI‑10 at 1 × 10^6^ cells·mL^−1^, supplemented with recombinant M‐CSF (for BMDMs) or GM‑CSF (for BMDCs) at 20 ng·mL^−1^, and plated in sterile tissue culture vessels. On days 3 and 5, half of the culture medium was gently removed and replaced with fresh RPMI‑10 containing 40 ng·mL^−1^ M‐CSF (for BMDMs) or GM‑CSF (for BMDCs). On day 7, cells were harvested for downstream applications.

### 4.13 Mouse Cell Stimulation

For stimulation assays, cells were plated at 1 × 10^6^ cells·mL^−1^ in fresh RPMI (or RPMI‐10 depends on cell type) and incubated at 37°C, 5% CO_2_. Cells were treated for 48 h with the test agent (CMCP) or vehicle control (Tag). After 48 h of stimulation, culture supernatants were collected for ELISA cytokine measurements.

### 4.14 Stimulation of Human Peripheral Blood Mononuclear Cells (hPBMCs) and qRT–PCR Analysis

Cryopreserved human peripheral blood mononuclear cells (hPBMCs, PB010C, Milecell Biotechnologies) were rapidly thawed in a 37°C water bath and immediately transferred into pre‐warmed RPMI 1640 medium supplemented with 10% fetal bovine serum (FBS) and 1% penicillin–streptomycin. Cells were washed by centrifugation at 500×*g* for 5 min to remove residual cryoprotectant and resuspended in complete RPMI 1640 medium supplemented with 1‰ β‐mercaptoethanol. For T cell activation and differentiation, cells were plated at 1 × 10^6^ cells·mL^−1^ and incubated at 37°C, 5% CO_2_, cultured in the presence of anti‐CD3 (2 µg·mL^−1^, 108567‐T08, Sino Biological China Inc.) and anti‐CD8 (2 µg·mL^−1^, 50389‐R208, Sino Biological China Inc.) antibodies. In addition, cells were treated for 48 h with the test agent (CMCP) or vehicle control (Tag). After 48 h of stimulation, cells were collected by centrifugation at 500×*g* for 5 min.

Total RNA was extracted from cells using Freezol reagent (R711, Vazyme Biotech Co., Ltd.) according to the manufacturer's instructions. The concentration and purity of RNA were determined by spectrophotometry (A260/A280 ratio). Quantitative real‐time PCR was performed using the HiScript II One Step qRT‐PCR SYBR Green Kit (Q221, Vazyme Biotech Co., Ltd.), following the manufacturer's protocol.

Relative gene expression levels were normalized to *UBE2D2* and calculated using the 2^−ΔΔCt^ method. All reactions were performed in triplicate.

### 4.15 *Apc^min/+^* Mouse Model of CRC

All animal experimentation related to the *Apc^min/+^* mouse model of CRC was approved by the Department of Health and Human Services and under protocols approved by the Ethics Committee of Institute of Microbiology, Chinese Academy of Sciences (IMCAS) and Institute of Zoology, Chinese Academy of Sciences (IOZCAS) (permit APIMCAS2022104, IMCAS and IOZ‐IACUC‐2024‐221, IOZCAS) in accordance with the NIH Guide for the Care and Use of Laboratory Animals, eighth Edition (2011). *Apc^min/+^* mouse were bred and maintained under SPF conditions for at least 1 week. All mice were fed with 60‐kcal%‐fat rodent diet (Research Diets, Inc) from 4 weeks old, maintained on a 12 h light/dark cycle encompassing 7 p.m. to 7 a.m., had free access to food and water, and were regularly monitored. Both males and females were treated and randomly distributed among groups. For elimination of irrelevant microbiota, broad‐spectrum antibiotics were administered. Neomycin (0.5 g·L^−1^) and ampicillin (1 g·L^−1^) were added in drinking water at 7 weeks for 5 days with plate‐spreading for sweeping effect validation. Antibiotics were withdrawn the day before EcN gavaging. Gavage was started at 8 weeks old and every 2 weeks thereafter. Chloramphenicol (50 mg·L^−1^) and C6‐HSL (10 µM) were added in drinking water. Stool samples were collected every week for strain colonization checking with PCR. Tumor‐bearing mice were euthanized with overall consideration of their conditions or at 20 weeks of age.

### 4.16 Tissue Collection and Image Analysis

Intestines were excised from all mice with mesenteric adipose tissue, and attached vessels were removed. A small portion of the duodenum was collected and immediately frozen in liquid nitrogen for RNA sequencing. The small intestine was separated from the colon, and the cecum was removed and discarded. The small intestine was evenly divided into three segments (proximal, mid and distal). Each segment was opened longitudinally, pinned flat on a wax plate, stained with 0.1% methylene blue, rinsed with ice‐cold phosphate‑buffered saline (PBS), and examined under a stereomicroscope; images were acquired. After inspection and imaging, segments were Swiss‑rolled, fixed in 4% paraformaldehyde for 24 h, transferred to 70% ethanol, and processed for embedding. Tissue embedding, sectioning, hematoxylin–eosin (H&E), and His‐tag immunofluorescence staining were performed by Beijing Bo Du Heng Yi Science & Technology Ltd. Anti‐His‐tag primary antibody (12698T, 1:800, Cell Signaling Technology) was applied. Serial sections were also subjected to multiplex immunofluorescence (mIF) staining. Tumor size and number were determined using Fiji software image analysis tools. Pathology scoring was followed according to a protocol by Erben et al. [99].

Multiplex immunofluorescence (mIF) was performed at the Pathological Platform of the Basic Medical Research Center, Peking University Third Hospital, to evaluate immune markers including CD3, CD8, CD19, CD45, CD38, and NK1.1. Formalin‑fixed paraffin‑embedded (FFPE) sections were dewaxed and rehydrated through successive xylene and graded ethanol baths and rinsed in distilled water. Antigen retrieval was then carried out by heat‑induced epitope retrieval (HIER) according to the manufacturer's instructions. (Pv‐6001, ZSGB‐Bio). After cooling, sections were washed in TBST and processed for sequential staining. Primary antibodies were applied as follows: rabbit anti‐CD45 (ab317446, 1:300, Abcam), rabbit anti‐CD19 (ab245235, 1:200, Abcam), CD3ε (E4T1B) rabbit anti‐CD3 (78588, 1:200, Cell Signaling Technology), CD8α (D4W2Z) rabbit anti‐CD8 (98941, 1:200, Cell Signaling Technology), rabbit anti‐CD38 (ab216343, 1:500, Abcam), NK1.1/CD161 (E6Y9G) rabbit anti‐NK1.1 (39197, 1:200, Cell Signaling Technology). HRP‐conjugated anti‐rabbit second antibody (Pv‐6001, ZSGB‐Bio) was used as the secondary antibody. 4′,6‐diamidino‐2‐phenylindole (DAPI) was used as a nuclear stain. The following reagents were used to detect secondary antibodies: Opal 480, Opal 520, Opal 570, Opal 620, Opal 690, and Opal 780 (Kuoran Biomedicine Technology (Shanghai) Co., Ltd.). Quantitative analysis was performed using Qupath (v0.6.0). For each experimental group, five of those samples were randomly selected for analysis.

### 4.17 RNA‐Seq Analysis

The qualified libraries were pooled and sequenced on Illumina platforms with PE150 strategy in Novogene Bioinformatics Technology Co., Ltd, according to effective library concentration and data amount. Cleaned sequencing data were downloaded and integrity‑checked using md5sum (v8.28). Reads were aligned to the mouse reference genome GRCm39 using HISAT2 (v2.2.1) [100]. Format conversion, sorting and mapping‑quality assessment were performed with SAMtools (v1.9) [91]. Transcript/gene read quantification was performed with featureCounts (v2.0.8) [101] using the appropriate annotation file. Differential expression analysis was carried out using DESeq (v1.46.0) together with GenomicFeatures (v1.58.0). Genes with adjusted *p‑value* (*padj*) < 0.05 and |log_2_fold change| > 1 were considered significantly differentially expressed. Volcano plots were generated with ggplot2 (v3.5.1). Pathway and functional enrichment analyses were performed with gene set enrichment analysis (GSEA) [102] using Gene Ontology Biological Process gene sets [103, 104, 105]. Visualization and enrichment workflows employed the clusterProfiler package (v4.10.1) and other R packages [106].

All data were expressed as the mean ± standard deviation (SD) or standard error of the mean (SEM) and analyzed using GraphPad Prism software (version 10.5.0). The statistical significance for all experimental groups was determined by two‐tailed unpaired Student's *t*‐test or ANOVA. A difference with a *p* < 0.05 was considered statistically significant. Significance is indicated directly in the figures with exact *p* values.

## Funding

National Key Research and Development Program of China (2025YFA1309200) and Open funding project of State Key Laboratory of Pharmaceutical preparation and delivery (No. 2023KF‐05).

## Conflicts of Interest

The authors declare no conflicts of interest.

## Supporting information




**Supporting File 1**: advs74820‐sup‐0001‐SuppMat.docx.

**Supporting File 2**: advs74820‐sup‐0002‐FigureS1.xlsx.

## Data Availability

The metagenomic datasets used in this study be accessed from the NCBI Sequence Read Archive (SRA) database under the accession number PRJDB4176, PRJEB10878, PRJEB12449, PRJEB27928, PRJEB6070, PRJEB7774, PRJNA397219, and PRJNA447983, while the proteomic datasets can be accessed from PRoteomics IDEntifications (PRIDE) Database under the accession number PXD000114, PXD003907, PXD005780, and PXD008870. The dataset generated for this study are available from the corresponding author upon reasonable request.
