# Supplementary material for: Identifying Cytokine Motif‐Containing, Immunomodulatory Bacterial Proteins in Human Gut Microbiome
Source: Adv Sci (Weinh). 2026 Mar 23;13(29):e20332. doi: 10.1002/advs.202520332 (PMC13205780; doi:10.1002/advs.202520332)
Supplement: Supplementary file 1 — Supporting File 1: advs74820‐sup‐0001‐SuppMat.docx. [file ADVS-13-e20332-s001.docx]

Supporting Information

Identifying cytokine motif-containing, immunomodulatory bacterial proteins in human gut microbiome

*Ziyu Wang, Siqi Guo, Jing Li, Qianqian Huang, Jing Ning, Xiang Liu, Zeyu Gao, Jin’e Li, Longchao Liu*, Moshi Song*, Jun Wang**

Z. Wang, Q Huang, L. Liu, J. Wang

a. Laboratory of Pathogen Microbiology and Immunology, Institute of Microbiology, Chinese Academy of Sciences, Beijing 100101, China

b. University of Chinese Academy of Sciences, Beijing 100049, China

Email: junwang@im.ac.cn

S. Guo

a. Laboratory of Pathogen Microbiology and Immunology, Institute of Microbiology, Chinese Academy of Sciences, Beijing 100101, China

Jing Li

c. Beijing Key Laboratory for Rheumatism Mechanism and Immune Diagnosis (BZ0135), Department of Rheumatology and Immunology, Peking University People’s Hospital, Beijing 100044, China

J. Ning

d. Beijing Key Laboratory for *Helicobacter Pylori* Infection and Upper Gastrointestinal Diseases (BZ0371), Department of Gastroenterology, Peking University Third Hospital; Beijing 100191, China

X. Liu

e. Laboratory of Microbial Resources and Biotechnology, Institute of Microbiology, Chinese Academy of Sciences, Beijing 100101, China

Z. Gao

f. State Key Laboratory of Organ Regeneration and Reconstruction, Institute of Zoology, Chinese Academy of Sciences, Beijing 100101, China

Jin’e. Li

e. Laboratory of Microbial Resources and Biotechnology, Institute of Microbiology, Chinese Academy of Sciences, Beijing 100101, China

b. University of Chinese Academy of Sciences, Beijing 100049, China

M. Song

f. State Key Laboratory of Organ Regeneration and Reconstruction, Institute of Zoology, Chinese Academy of Sciences, Beijing 100101, China

g. Beijing Institute for Stem Cell and Regenerative Medicine, Beijing 100101, China

b. University of Chinese Academy of Sciences, Beijing 100049, China

**Table S1.** **Protein candidate chosen in this study**

| CMCP | Length | Sequence |
| --- | --- | --- |
| bCCL8 | 227 aa | QVTRENRIPTRLPAITTWIIPGILIPSSICRQPNRTDGAITAIFRFHAYSTELIIIPRNTVSSTIPVANPKYATLPATSCFNVSASKVDCCHVPANNWYIRITNSGIAKAAMIQNGYRSGFPILKSAIGIFNIFLQIRKTPPSTRPTYKIPVKIFPVTADIPPATSIFPIRSAKSCENARRQSARILTPAAFVPLNFVCLLLLISAPFSFAGKCSDLFYISNRFISV |
| bIFNL3 | 125 aa | ICLMMALLLYRSAKPAANRDDAERKEWLRQQLEAQNRQMDAKLAEMAQQNLAAMGHISETLQASVQSMSTALAAGQGTQQQTMEQRLQGLEASNARKLEEMRRTLAEGMTALQAQNAQKLDEIRL |
| bIL1 | 480 aa | TTENTAAHNDIDPTVWVETVDGEEHRYLAWGNTLYYVCELNEDMTSVKDINGDGIVDKKDVKLQTINNLPAGLGYTEAPWIYRRTDENGNYTGKYYLFAAFGWREQMAYATSDTMWGPWEFGGVLMPPTATSNTNHPSVIDFKGKTYFIYHNGSKPWGSGFRRVVCAEEFTINDDGTIDPIQETSTGLTGTKSAIMQNGGYIYHDNFVNPSDDASYPLVKNVYFGKRYSASKDTQWEIVAGKADTANENYVSIAAVNKPGLYIAARSDNSVILTQDSKQNDTSMQKAMTFKTVKGLNGQENAVSFESVLKPGYYLSTKDSAITLISCSDIDRETSSFEIGAALGDDSISGMMADISGAWIKNGSSVRFYLNNAALYQNVNAYVAEYKDGMLIGVGAVNNIEINSSIQSIEIPYERKDNDSELKIFVWNSMLPATEPVNVTVMENPYAMPTGYTSYFNFDENINDTQTEAQGSLVGAKITD |
| bIL12 | 402 aa | SINCEIYHVMTKKSPPMTNIYGIKTKTMLLLYKYRIKPVIKQRLFSNLALIGVKNKRRKQMIKTIMKRCAVLMLCMVMTVGLFGCNLSVNNGKRIVRIAIAQSETHPEYLGLVAFKEYVEERLGDKYEVQIFPNELLGSIQKTIELTQTGAIDFAVAGTANLETFADVYEVFSMPYLFDSVESYKAVMQDTDYMENVYESTDSSGFRVLTWYNAGTRNFYGKKAINTPADLSGMKIRVQQSPASVAMMQAFGAAASPMSFGEVYTAIQQGVIDGAENNELALTNNKHGEVAKYYTYNMHQIIPDMLVGNLKFIQGLDEDELKVFKEAALKSTEVELTEWDKCVEEAKNTAHNEMGVEFIYPDITLFKDKVKNMQQDMIQKNPSIVDIYNHIQEVNKRIGEEK |
| bIL33 | 137 aa | EIANEYALDYLSETKLRRIELEMKSLSTFNDTRIMFITPSGDVILDTNDSSTDKSNDDRVLFSINDFDYGDLKGKHDILWDFYGLFSEPALSVFSPISNSFEIKGYVVINIPESAIVERVYDTFNTNYLTLAIVLIL |
| bLECT2 | 101 aa | RHGAGIFGHWAGHGVCGRYFRLAGGWRAGRCGRWCGPVAETTPGSAERLPGQAYFCRAGPRKPGSGRYRLPAEQGGYGHRNRRIDRNRPLGRACVCAKLLE |
| bMIF | 124 aa | NILNKNKEEKYMPFISFTTNHKLTLRQENEIAKRTGELITILPGKKEENLMLHLEDNQIMYFRGDDIPCMMIAVKLYNTIDFDAKKKFTEELVKMIKETTNIEINDVYVSFDEYPNWGKQGTLF |
| bOPN | 149 aa | DSYLFDLSETSDYSLIPPIVLIAPIVLIAPIAQTPPITNSQPQKNKTMDAVTSDTTDAQI  VRPYRETYHSYSSDSSDRFNRSNTSDHSDHSDHSDTLRLLRPTQIPPMTLIAPITPITNS  QQHKKRVPRQVSYLSEDSPMGKAVTYSPD |

**Table S2. Summary of HMM-based motif detection in representative human cytokines.**

| CMCP | HMM motif length (aa) | Aligned region in human reference protein (aa) | Functional domain involved | Key functional sites covered | Interpretation |
| --- | --- | --- | --- | --- | --- |
| bCCL8 | 99 | 1–99 (full length) | Canonical chemokine fold | CC motif; receptor-binding core | Complete conservation of canonical chemokine domain |
| bIFNL3 | 203 | 1–196 (~full length) | Type III interferon α-helical bundle | IFNLR1 / IL10R2 receptor-binding interface | Full conservation of functional cytokine fold |
| bIL1 | 119 | Core β-trefoil domain | IL-1/IL-36 structural fold | Receptor interaction surfaces | Canonical IL-1 family structural domain detected |
| bIL12 | 214 | 7–219 (full length) | IL-12 α subunit cytokine fold | IL-12 receptor interaction region | Conserved heterodimeric cytokine subunit |
| bIL33 | 158 | 114–268 (mature domain) | IL-1-like β-trefoil fold | ST2 receptor-binding interface | Conserved IL-1 superfamily cytokine fold |
| bLECT2 | 96 | 52–140 | Peptidase_M23-like fold | Structural core only | Structural similarity |
| bMIF | 114 | 2–115 (full length) | MIF tautomerase fold | Catalytic Pro1 region | Canonical MIF enzymatic cytokine domain conserved |
| bOPN | 291 | 66–133 (partial) | Asp/Ser-rich low-complexity region | RGD integrin-binding motif NOT detected; CD44-binding region not covered | Similarity restricted to low-complexity region; no conservation of major functional motifs |


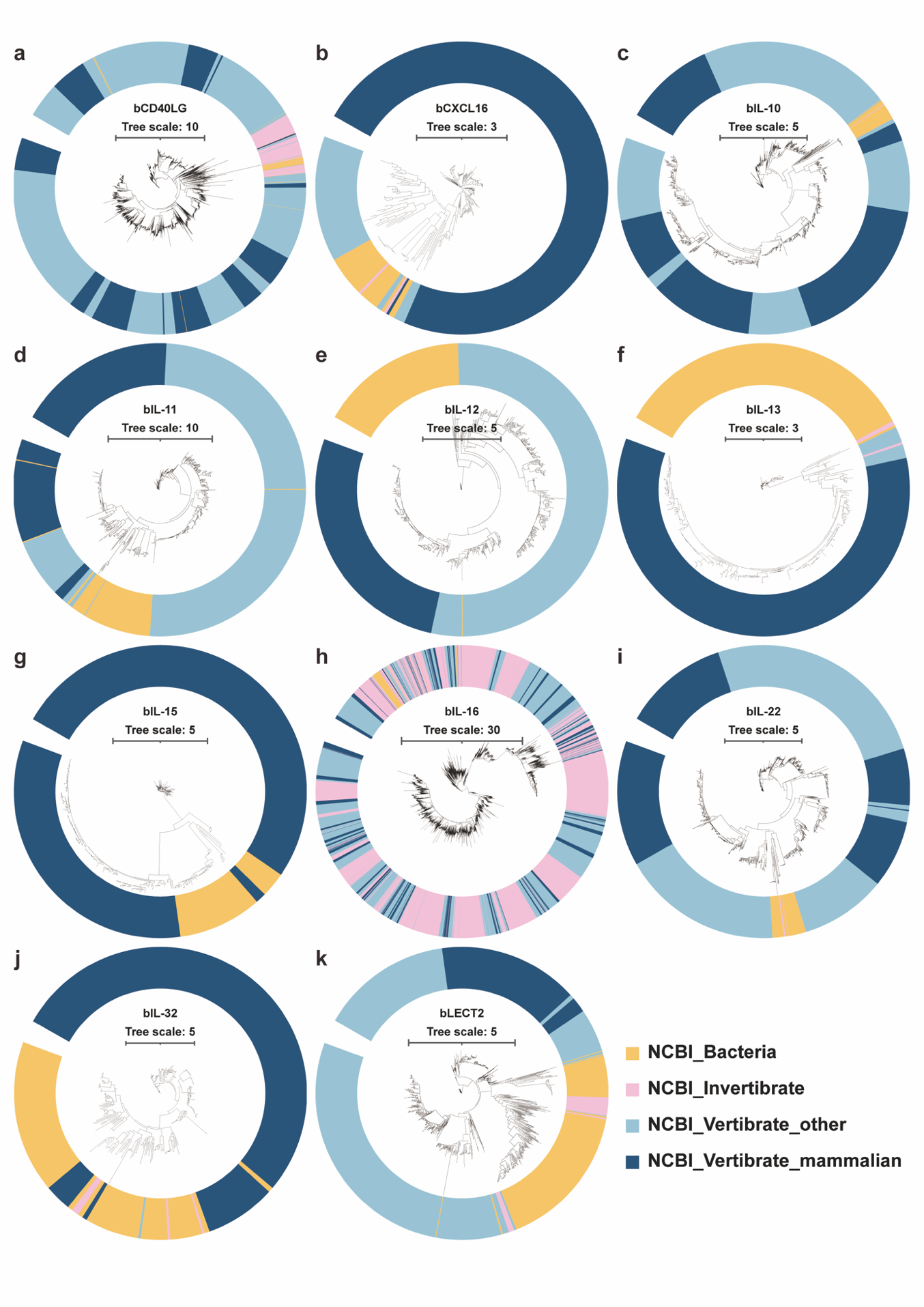


Figure S1. Distribution of top CMCP hits across different genome databases. For each CMCP sequence, the tree end points indicate its origin (Bacteria genomes (orange); invertebrate genome (pink); mammalian vertebrates (dark blue) vertebrate others (light blue)). a. bCD40LG, b. bCSCL16, c. bIL-10, d. bIL-11, e. bIL-12, f. bIL-13, g. bIL-15, h. bIL-16, i. bIL-22, j, bIL-32. k bLECT2.

Figure S2. Frequency of occurrence of the top 10 CMCPs in human fecal proteome.


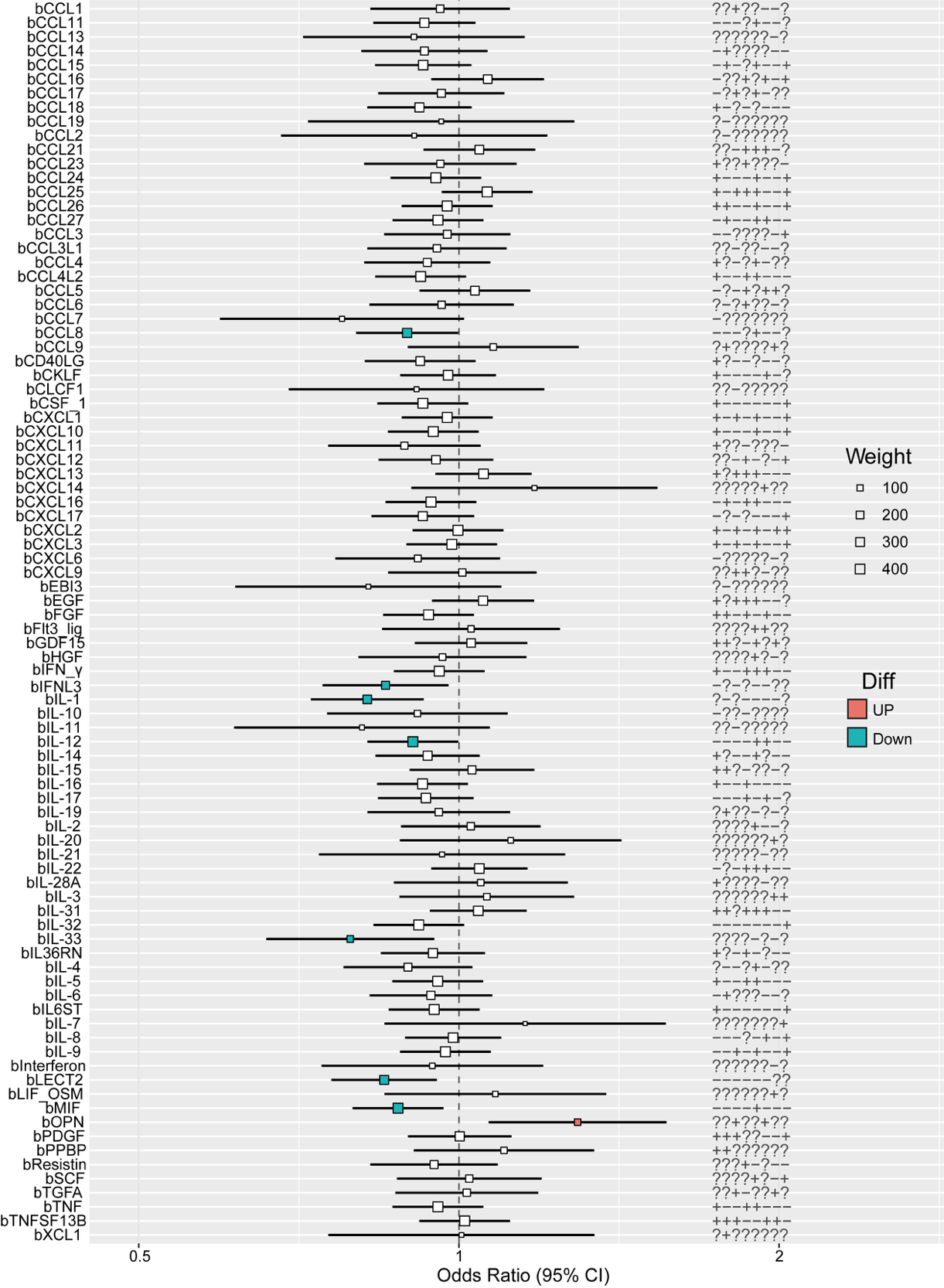


Figure S3. Enrichment results for meta-analysis of CRC-related metagenome mining of CMCPs (Only significant CMCPs included). X‑axis indicates odds ratio; horizontal error bars represent the 95% confidence intervals. The Y‑axis lists the eight enriched cytokines. Red boxes denote higher abundance of the cytokine homologs in colorectal cancer (CRC) patients, whereas blue boxes denote lower abundance in CRC patients (i.e., relatively enriched in healthy controls). Box area represents the enrichment weight of the homologs. Symbols on the right “+”, “−”, “?” indicate this CMCP is significantly enriched in CRC patients, significantly depleted in CRC patients (enriched in healthy controls), or not significant, respectively in each cohort. Cohorts are ordered left to right as follows: PRJDB4176, PRJEB10878, PRJEB12449, PRJEB27928, PRJEB6070, PRJEB7774, PRJNA397219 and PRJNA447983. CCL4L2, C‑C motif chemokine ligand 4‑like 2; CLCF1, cardiotrophin‑like cytokine factor 1; CRC, colorectal cancer; Ctl, control; CSF, colony‑stimulating factor; CXCL, C‑X‑C motif chemokine ligand; EBI3, Epstein–Barr virus induced 3; EGF, epidermal growth factor; FGF, fibroblast growth factor; FLT3 ligand, FMS‑like tyrosine kinase 3 ligand; HGF, hepatocyte growth factor; HMM, hidden Markov model; IFN, interferon; IFNL3, interferon lambda 3; IL, interleukin; IL36RN, interleukin 36 receptor antagonist; IL6ST, interleukin‑6 signal transducer; LECT, leukocyte cell‑derived chemotaxin; LECT2, leukocyte cell‑derived chemotaxin 2; LIF/OSM, leukemia inhibitory factor / oncostatin M; MIF, macrophage migration inhibitory factor; PDGF, platelet‑derived growth factor; PPBP, pro‑platelet basic protein (CXCL7); SCF, stem cell factor; TGFA, transforming growth factor alpha; TNF, tumor necrosis factor; TNFSF13B, tumor necrosis factor (ligand) superfamily member 13B; XCL, X‑C motif chemokine ligand.


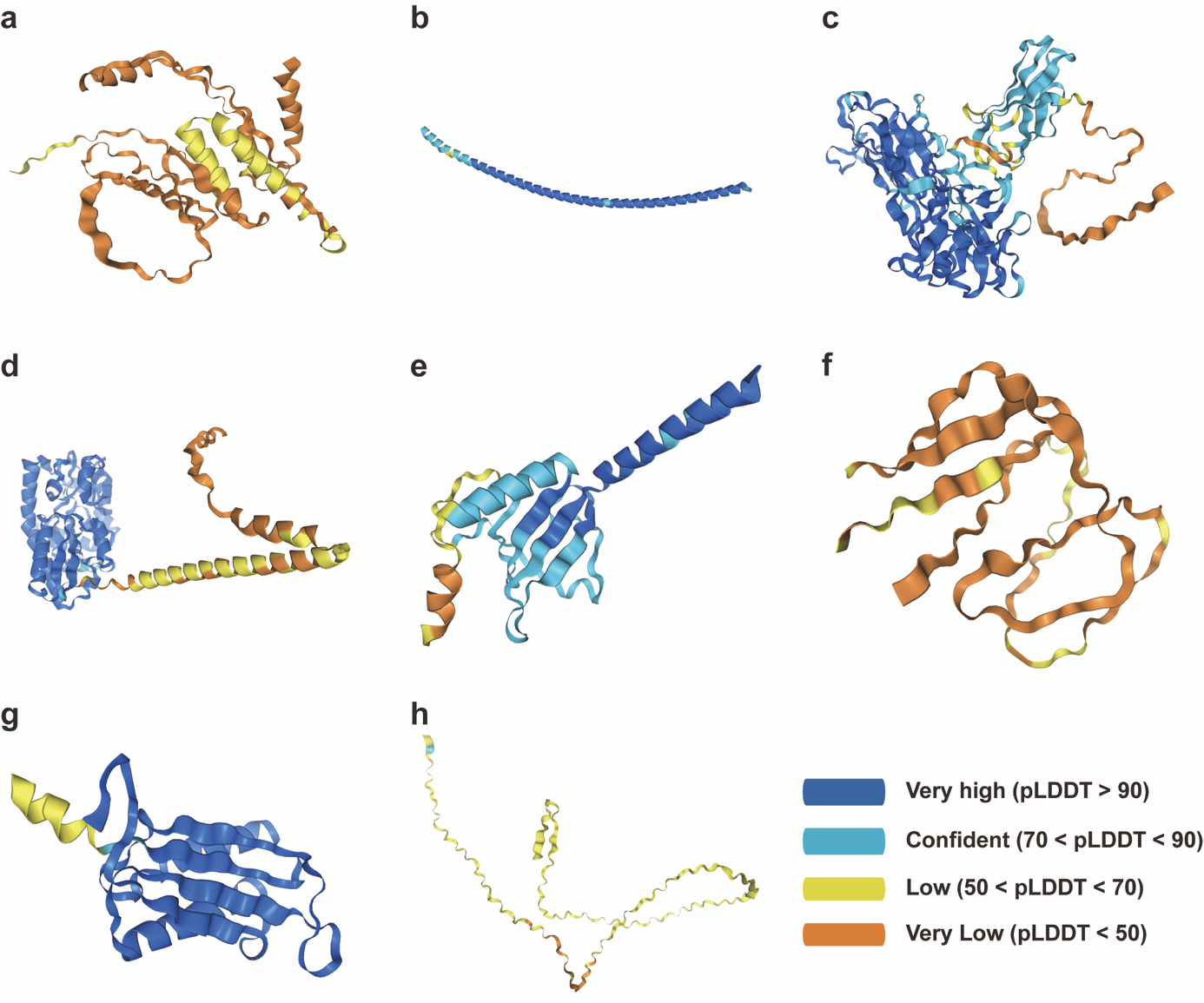


Figure S4. AlphaFold 3 predicted CMCP candidate structures. The labels are as follows: a. bCCL8, b. bIFNL3, c. bIL-1, d. to bIL-12, e. bIL-33, f. bLECT2, g. bMIF, and h. bOPN. The colors represent ipTM, with the orange portions indicating low prediction accuracy (ipTM < 50), the yellow sections denoting very low prediction accuracy (50 < ipTM < 70), the light blue areas reflecting low prediction accuracy (70 < ipTM < 90), and the dark blue regions representing very low prediction accuracy (ipTM > 0).

Figure S5. Characterization of EcN density after induction of EcN-I-CMCP. After induced by C6-HSL, OD_600_ was measured to quantify EcN density. Data are presented as mean ± SD. Statistical analysis was performed by mixed effect analysis (factors: treatment and time) followed by multiple comparisons using Turkey’s test. Significance is indicated as **P* < 0.05 and ***P* < 0.01.


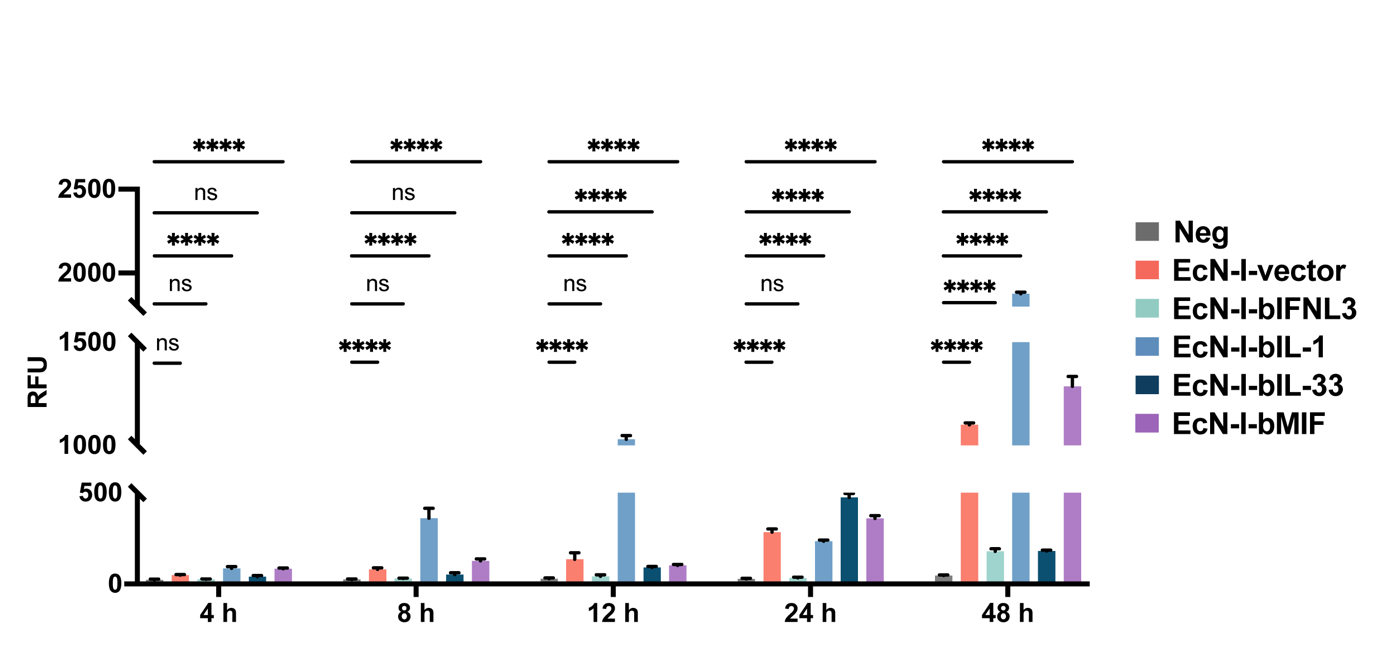


Figure S6. Characterization after induction of EcN-I-CMCP. After induced by C6-HSL, cultures were collected and relative fluorescence units (RFU) levels were measured. Data are presented as mean ± SD. Statistical analysis was performed by two‑way ANOVA (factors: treatment and time) followed by multiple comparisons using Dunnett’s test. Significance is indicated as *****P* < 0.0001 and ns, no significance.

Figure S7. Quantification of CMCPs released by EcN-I-CMCP in *Apc^min/+^* mouse intestine revealed by anti-His-tag immunostaining. a. The production of CMCPs in EcN-I-bIL-1 and EcN-I-bIL-33 strains measured by His-tag ELISA. b. Relative fluorescence intensity of CMCP in proximal intestinal sections of *Apc^min/+^* mice following treatment with engineered bacteria. c. The representative immunostaining images of proximal intestinal sections from *Apc^min/+^* mice following treatment with engineered bacteria. First column: 5× objective; second column: 35× objective. Scale bars: 200 μm (first column), 50 μm (second to fourth column).

Figure S8. Proportion of CD45^+^ cells, CD45^+^CD3^+^cells, CD45^+^CD19^+^cells and CD45^+^CD3^-^NK1.1^+^cells in tumor region relative to the paratumor region following treatment with engineered bacteria. Quantification was performed on n = 5 mice per group, with 5 independent fields per mouse. Cells were counted and normalized to area. Statistical analysis was performed by one‑way ANOVA followed by multiple comparisons using Dunnett’s test. Data are presented as the mean ± SEM, *****P* < 0.0001, ns, no significance.


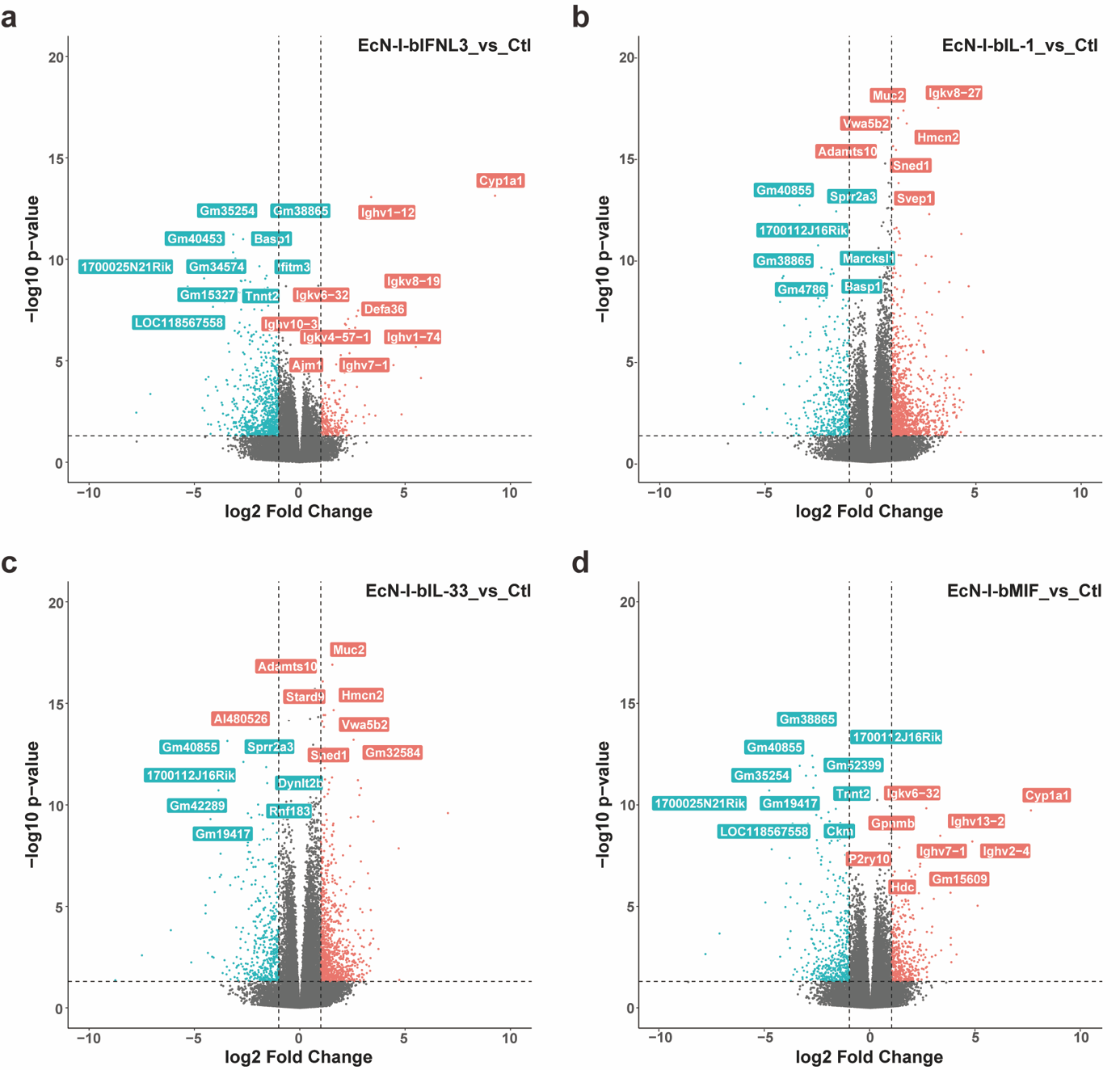


Figure S9. Volcano plots of genes enriched in different treatment groups. The x-axis in this figure represents the log2 fold change in gene expression, while the y-axis represents the -lg significance of the difference. Two vertical dashed lines in the figure represent the threshold at |FC|=2, (|±log2(2) | = 1), and the horizontal dashed line represents the threshold at *P* = 0.05, (-lg (0.05) = 1.3). Red indicates up-regulated genes, blue indicates down-regulated genes and gray represents no significant difference. Labels a-d correspond to each treatment group: a. EcN-I-bIFNL3 vs Ctl, b. EcN-I-bIL-1 vs Ctl, c. EcN-I-bIL-33 vs Ctl and d. EcN-I-bMIF vs Ctl.

Figure S10. Recombinant CMCPs activate hPBMC in vitro. a-e. Relative cytokine mRNA expression levels in different treatment groups after CMCP treatment during CD3/CD28-induced T cell activation in hPBMCs. Statistical analysis was performed by one‑way ANOVA followed by multiple comparisons using Turkey’s test. Data are presented as the mean ± SEM, **P* < 0.05, ***P* < 0.01, ****P* < 0.001. a. *IFNG*, b. *IL6*, c. *IL10*, d. *IL17A*, e. *TNF*.

Figure S11. CMCP binding test with corresponding classical cytokine receptor. a-c. Representative BLI sensorgrams showing the association and dissociation phases of CMCP (and corresponding tagged proteins) binding to immobilized corresponding classical cytokine receptor at three analyte concentrations: a, 100 nM; b, 10 µM; c, 70 µM. Kinetic parameters were obtained by fitting the sensorgrams using a 1:1 Langmuir binding model. d-g. Luciferase reporter assay measuring receptor-specific activation by CMCPs. d. IL1R1 reporter activation by rhIL-1β. e. IL1R1 reporter activation by DsbA-Tag and DsbA-IL1, respectively.

g. IL1RL1 (ST2) reporter activation by rhIL-33. i. IL1RL1 (ST2) reporter activation by MBP-Tag and MBP-IL33, respectively.
